# Activity-Based Protein Profiling of RHBDL4 Reveals Proteolysis of the Enzyme and a Distinct Inhibitor Profile

**DOI:** 10.1021/acschembio.4c00273

**Published:** 2024-07-23

**Authors:** Cassondra C. Davies, Ren-Ming Hu, Paul J. Kamitsuka, Gabriel N. Morais, Regina Stasser de Gonzalez, Katelyn A. Bustin, Megan L. Matthews, William H. Parsons

**Affiliations:** † Department of Chemistry and Biochemistry, Oberlin College, Oberlin, Ohio 44074, United States; ‡ Department of Chemistry, University of Pennsylvania, Philadelphia, Pennsylvania 19104, United States

## Abstract

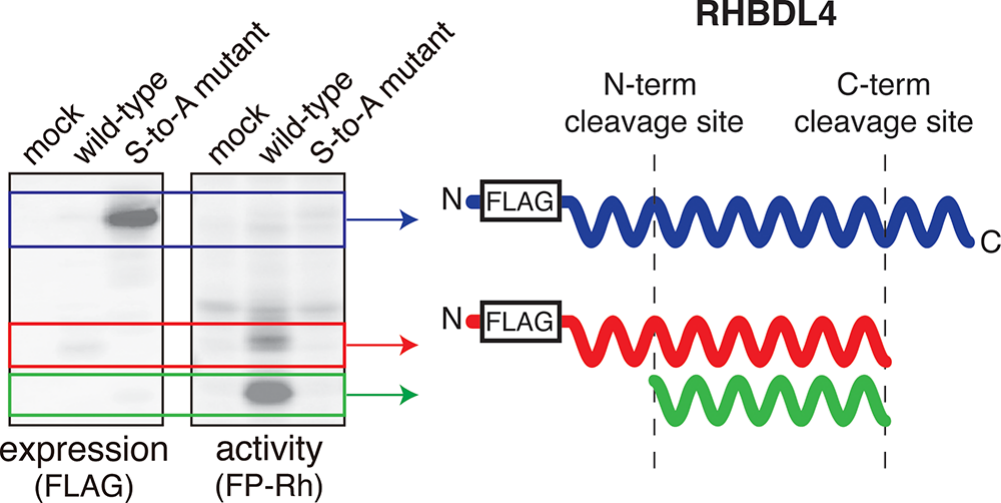

Rhomboid proteases have fascinated scientists by virtue of their membrane-embedded active sites and proposed involvement in physiological and disease pathways. The human rhomboid protease RHBDL4 has generated particular interest due to its role in endoplasmic reticulum-associated protein degradation and upregulation in several cancers; however, chemical tools for studying this enzyme are currently lacking. Here, we describe the development of an activity-based protein profiling (ABPP) assay for RHBDL4. We have employed this assay to determine that human RHBDL4 undergoes proteolytic processing in cells to produce multiple active proteoforms with truncated C-termini. We have also used this assay to identify chemical scaffolds capable of inhibiting RHBDL4 activity and have observed distinct inhibitor preferences between RHBDL4 and a second human rhomboid protease PARL. Our work demonstrates the power of ABPP technology to characterize active forms of enzymes that might otherwise elude detection and the potential to achieve selective inhibition among the human rhomboid proteases.

Nature has developed a host of complementary methods to promote the hydrolysis of chemical bonds. Among the numerous enzymes that catalyze hydrolysis reactions, rhomboid proteases have generated significant interest due to their membrane-embedded active sites.^1^ This structural feature appears to be evolutionary advantageous as members of this subset of serine proteases have been identified in all kingdoms of life.^2^ Following the initial characterization of a rhomboid protease in 2001,^3^ research conducted over the past two decades has provided important insight into these enzymes.

Five human rhomboid proteases have been identified to-date: rhomboid-related proteins 1–4 (RHBDL1–4) and presenilin-associated rhomboid-like protein, mitochondrial (PARL). Among these, RHBDL4, which is encoded for by the rhomboid domain-containing 1 gene (RHBDD1), has sparked particular interest due to its association with the endoplasmic reticulum-associated degradation (ERAD) pathway as well as multiple diseases.^4,5^ Upregulation of RHBDL4 has been observed in breast cancer, nonsmall cell lung cancer, and colorectal cancer.^6−9^ Knockdown or knockout of RHBDL4 has been shown to reduce cell growth and promote cell apoptosis in these cancers, which suggests that functional inhibition of RHBDL4 may be therapeutically beneficial. At the same time, the underlying biology of RHBDL4 is complex and not fully delineated. A study^10^ to capture substrates of RHBDL4 uncovered 25 candidate substrates for the enzyme with additional substrates described elsewhere in the literature.^11−14^

Despite their potential ability to aid ongoing studies of the rhomboid proteases, chemical tools to probe the biology of these enzymes, including RHBDL4, are still limited. To address this gap, several groups have employed activity-based protein profiling (ABPP) technology as a strategy to study these enzymes.^15−17^ At the outset of this work, we postulated that an ABPP assay, employing a chemical probe that engages functional, but not inactive, forms of RHBDL4, would represent an attractive platform for monitoring RHBDL4 activity. The use of this methodology with other rhomboid proteases has provided insight into catalytically important residues^15^ and furnished a platform for discovering inhibitors.^16^ We were particularly interested in using ABPP to identify the active form(s) of RHBDL4 as prior studies of RHBDL2 and PARL indicate that these proteins may undergo N-terminal proteolytic processing to generate their mature active forms.^18,19^

Here, we demonstrate that fluorophosphonates serve as effective activity-based probes for RHBDL4 and enable detection of multiple active forms that, to the best of our knowledge, have not been previously characterized. Through LC-MS/MS-based proteomics and protein mutagenesis, we determined that heterologously expressed RHBDL4 undergoes extensive proteolytic processing of its large cytosolic C-terminal domain in cells. Truncated forms of RHBDL4 both react with our activity-based probe and cleave amyloid precursor protein (APP), one of the enzyme’s reported substrates. By employing our ABPP assay in a competitive format, we also identified chemical scaffolds capable of inhibiting RHBDL4 activity and observed differences in the inhibitor preferences of RHBDL4 and PARL. Collectively, our findings demonstrate the multidimensional nature of activity-based probes as tools to both uncover previously undescribed biology and facilitate the development of enzyme inhibitors.

In seeking suitable activity-based probes for RHBDL4, we first investigated fluorophosphonates based on their commercial availability and selective reactivity with serine hydrolases (Figure 1A).^20^ To explore the potential of a fluorophosphonate (FP) probe to label active RHBDL4, we accessed constructs encoding for expression of wild-type (WT) mouse RHBDL4 (mRHBDL4) and human RHBDL4 (hRHBDL4) with either N-terminal or C-terminal epitope tags (Myc and FLAG). We also generated inactive mutants with the nucleophilic Ser144 mutated to an alanine. 48 h after transfection of HEK293T cells with these constructs, we lysed the cells and pelleted the membrane fraction by ultracentrifugation. We refer to the resuspended membrane fraction as the membrane proteome of the cells. Each membrane proteome was then treated with a fluorescent FP probe (FP-Rh) and separated by SDS-PAGE. After collecting an in-gel fluorescence measurement to detect probe-labeled bands, we transferred the proteins to a nitrocellulose membrane and then detected bands containing the FLAG epitope tag with an anti-FLAG antibody.

**Figure 1:**
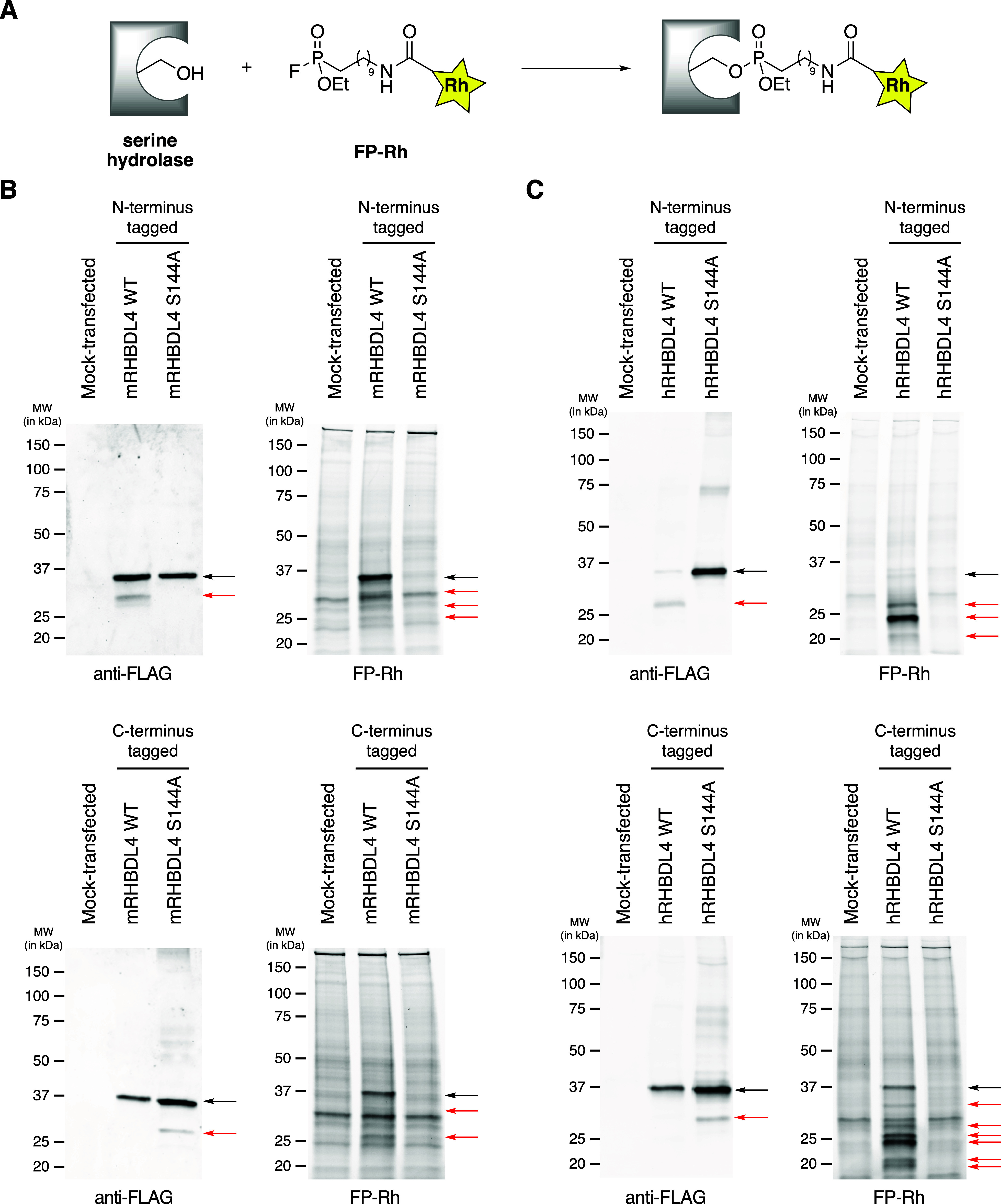
FP-Rh labels mouse and human RHBDL4 in an activity-dependent manner. (A) General reaction of a serine hydrolase with the FP-Rh probe. (B) Western blots and ABPP gels for membrane proteomes of HEK293T cells transfected with empty vector (“Mock”), wild-type (WT) mouse RHBDL4 (mRHBDL4), or the S144A mutant. (C) Western blots and ABPP gels for membrane proteomes of HEK293T cells transfected with empty vector (“Mock”), wild-type (WT) human RHBDL4 (hRHBDL4), or the S144A mutant. Constructs encoding for either N-terminal epitope tags or C-terminal epitope tags were used as indicated. Black arrows indicate bands consistent with full-length RHBDL4; red arrows indicate RHBDL4-associated bands at lower molecular weights.

For the membrane proteomes of cells transfected with wild-type and mutant mRHBDL4, we observed prominent bands at the expected molecular weight (∼36 kDa) for the full-length protein in the Western blots for both N- and C-terminal tagged variants (Figure 1B, *left*). We also observed corresponding fluorescent bands for wild-type mRHBDL4 in the associated ABPP gels (Figure 1B, *right*). These fluorescent bands are absent for the inactive S144A mutant, confirming that the nucleophilic serine is essential for probe labeling. In a related experiment, we observed that probe labeling of mRHBDL4 is time-dependent (Supplemental Figure 1), consistent with a covalent reaction between the probe and the enzyme. Due to the broad reactivity of the FP-Rh probe, other fluorescent bands are observed in the ABPP gel as can be seen in the mock-transfected control lanes. However, we also observed lower molecular weight bands in the ABPP gel that only appear in the wild-type mRHBDL4 lanes, which suggests that these represent truncated forms of the enzyme.

When we repeated the same analysis for cells transfected with wild-type and mutant hRHBDL4, we observed notable differences. While the S144A mutants produce robust bands at the expected molecular weight of the full-length protein in the Western blots, the bands corresponding to the full-length protein are weaker for the wild-type enzymes with the full-length N-terminal tagged variant nearly undetectable (Figure 1C, *left*). A Western blot employing a commercial anti-RHBDL4 antibody, generated using an epitope corresponding to amino acids 209–313, produced similar results (Supplemental Figure 2). In the corresponding ABPP gel, we observed several new fluorescent bands at molecular weights below 36 kDa (Figure 1C, *right*). Many of these fluorescent bands, including the most prominent bands for both N-terminal and C-terminal tagged hRHBDL4, lack corresponding bands in the Western blot. None of the new fluorescent bands observed for wild-type hRHBDL4 are produced by the S144A mutant, indicating that they all require the presence of the nucleophilic serine.

RHBDL4 appears to undergo proteolysis based on these results. The lower molecular weight bands observed in the ABPP gels for both C-terminally tagged mRHBDL4 and hRHBDL4 lack corresponding bands in the Western blots, indicating loss of the epitope tag. In the case of the N-terminally tagged proteins, one of the prominent lower molecular weight bands in the ABPP gels is also observed in the Western blots. However, the most prominent band in the ABPP gel for N-terminally tagged hRHBDL4 has no corresponding band in the Western blot, which suggests that the epitope tag has been lost. Collectively, these data indicate that proteolysis of RHBDL4 occurs at both its N- and C-termini and that these truncated forms retain activity based on their reactivity with the FP probe. In addition, the notable differences in the bands observed for wild-type hRHBDL4 and the inactive mutant in the Western blots suggest that processing may, at least in part, be dependent on the enzyme’s activity.

To avoid their potential impact on FP-Rh labeling, we initially omitted protease inhibitors when preparing the membrane proteomes. To investigate whether the lower molecular weight bands were due to proteolytic events postlysis, we compared whole cell lysates from cells lysed in the presence and absence of a protease inhibitor cocktail and then treated with FP-Rh. We found that the presence of a protease inhibitor cocktail did not alter the pattern of bands observed for hRHBDL4 (Supplemental Figure 3). We further explored the possibility of proteolysis postlysis by incubating both hRHBDL4 and mRHBDL4-transfected membrane proteomes for up to 4 h at 37 °C prior to FP-Rh treatment. We observed similar band patterns at all time points for both hRHBDL4 and mRHBDL4 (Supplemental Figure 3). The results of these experiments suggest that proteolysis of the enzyme to produce lower molecular weight forms is most likely a cellular event.

Proteolyzed forms of heterologously expressed RHBDL4 have been previously observed in Western blots,^14,21^ though the molecular details and catalytic activities of these forms have not been characterized. Notably, several of the truncated forms of RHBDL4 we observed are robustly detected with the activity-based probe but not by Western blot and may therefore have eluded detection in prior studies. Among the human rhomboid proteases, the most well-characterized proteolytic processing events are associated with PARL, which undergoes multiple N-terminal cleavage events to generate forms that have been detected for both overexpressed and endogenous enzyme.^18^ It has also been proposed that RHBDL2 undergoes N-terminal processing to adopt its active form.^19^ Processing of RHBDL4 would therefore not be unprecedented among the human rhomboid proteases, though C-terminal cleavage is unique compared to the cases that have been previously described.

To gain further insight into the observed proteolytic events, we conducted mass spectrometry-based proteomics experiments to investigate the sequences of the truncated forms. Enrichment of membrane proteomes generated from HEK293T cells overexpressing hRHBDL4 with a biotin-tagged fluorophosphonate probe (FP-biotin)^22^ produced multiple RHBDL4-derived peptides, providing additional evidence for covalent labeling of the enzyme (Supplemental Table 1). However, the detection of peptides spanning most of the sequence made it difficult to use these data to gain insight about potential sites of proteolysis.

We therefore turned our attention to characterizing the lower molecular weight bands observed for N-terminally tagged hRHBDL4. We excised each of the bands observed in our ABPP gel and digested them with trypsin (Figure 2A). Based on our initial Western blots and ABPP gels, we hypothesized that the enzyme undergoes cleavage at its C-terminus to produce the band at ∼27 kDa (“band 2”) and cleavage at both the N- and C-termini to produce the band at ∼23 kDa (“band 3”). Despite the technically challenging nature of this experiment, we detected multiple peptides for each band and observed an increased number of spectral counts for band 3, which was most prominent in our ABPP gel (Figure 2B). Though the limited number of trypsin cleavage sites near the N-terminus of RHBDL4 impacted detection of peptides at the beginning of the sequence, we saw robust detection of peptides in the interior of the sequence (amino acids 138–260). Most notable were the differences observed at the C-terminus for the three isolated bands. While band 3 produced the highest spectral counts for most of the detected peptides, comparatively low spectral counts were obtained for peptides spanning amino acids 260–285, and the peptide corresponding to amino acids 290–305 was not detected at all. Based on these results, we hypothesized that truncation at the C-terminus might occur where detection of tryptic peptides decreases. We also performed the in-gel digestion experiment using chymotrypsin. While the total number of detected peptides was lower for all three bands in this experiment, we detected two peptides near the N-terminus (amino acids 21–32 and 41–49) for bands 1 and 2 but not band 3 (Supplemental Table 2). We similarly used these data to inform further investigation of a potential site of truncation at the N-terminus.

**Figure 2:**
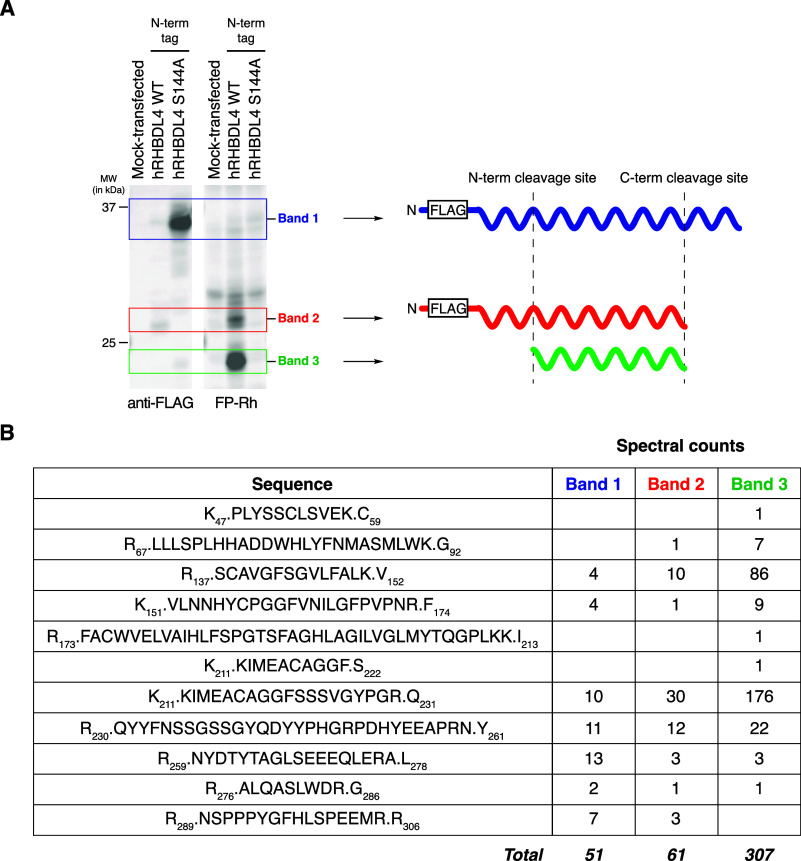
In-gel digestion and analysis of the proteolyzed forms of human RHBDL4. (A) Western blot and ABPP gel images for hRHBDL4-transfected HEK293T membrane proteomes used to define band positions for in-gel digestion experiments with cartoon of proposed cleavage events that give rise to the observed bands. (B) Spectral counts for the tryptic peptides obtained for each band.

Based on these results, we generated a series of hRHBDL4 deletion mutants to investigate whether shortened forms of the enzyme retain activity. Using the peptides observed in our proteomics experiments and the predicted structure of hRHBDL4 (AlphaFold Q8TEB9^23^) to guide our mutations, we constructed mutants truncated at each terminus (Figure 3A). We observed that all four C-terminal deletion mutants were successfully expressed (Figure 3B). Furthermore, all four of these mutants are labeled by FP-Rh with two mutants (Δ261–315 and Δ278–315) producing bands highly similar to those observed for the wild-type enzyme, suggesting that these amino acids are absent in the most prominent forms visualized in our ABPP gel. This finding supports our hypothesis that hRHBDL4 undergoes C-terminal proteolytic cleavage in cells. While the other two mutants produce different sets of bands, it is noteworthy that the removal of nearly a third of the sequence generates a form of the enzyme that can still be engaged by the probe.

**Figure 3:**
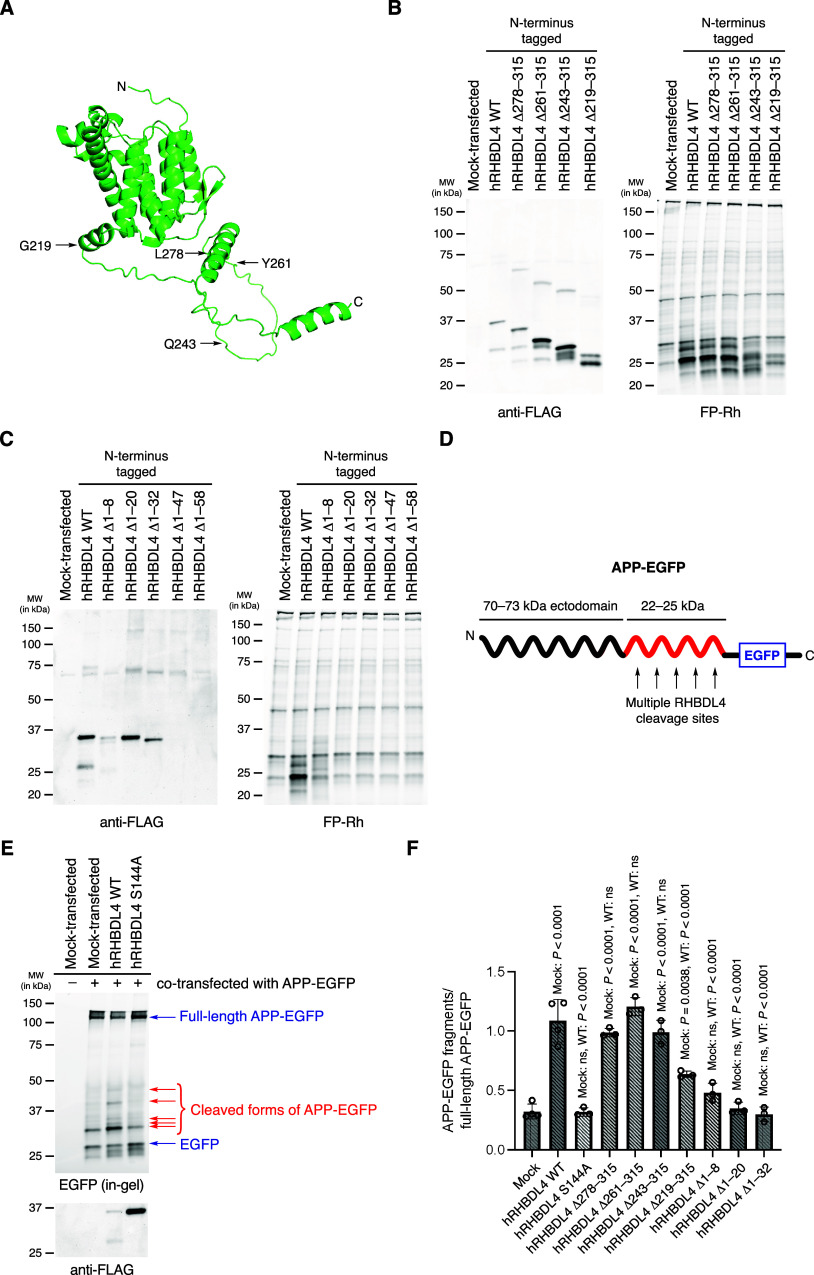
Truncated forms of human RHBDL4 retain catalytic activity. (A) AlphaFold predicted structure (AF-QETEB9-F1^23,26^) of hRHBDL4 with sites of C-terminal truncation indicated by arrows. The ribbon diagram was generated by PyMol Molecular Graphics System (v.2.6.0). (B) Western blot and ABPP gel for the whole cell lysates of HEK293T cells transfected with empty vector, wild-type hRHBDL4, or the indicated C-terminal deletion mutant. (C) Western blot and ABPP gel for the whole cell lysates of HEK293T cells transfected with empty vector, wild-type hRHBDL4, or the indicated N-terminal deletion mutant. (D) Cartoon of APP-EGFP fusion showing region of proposed RHBDL4-mediated cleavage events. (E) Representative in-gel fluorescence image and Western blot of the whole cell lysates of HEK293T cells cotransfected as indicated. Arrows are used to indicate the positions of the full-length APP-EGFP fusion, EGFP fused to APP fragments, and EGFP alone. (F) APP proteolysis activity of HEK293T cells cotransfected with the APP-EGFP fusion and the indicated RHBDL4 construct. Activity was quantified by determining the ratio of the bands between 30–50 kDa and the band for the full-length protein. Data represent the mean ± standard deviation for *n* ≥ 3 independent experiments. *P* values were determined by one-way ANOVA and posthoc Tukey tests with each column compared to the mock-transfected and hRHBDL4 WT-transfected results (ns = not statistically significant).

We found that only a subset of our N-terminal deletion mutants produced bands in a Western blot (Figure 3C). For the three mutants that could be detected, the Δ1–8 mutant gives rise to faint bands in the corresponding ABPP gel, but no FP-labeled bands above background could be detected for the Δ1–20 or Δ1–32 mutants. Given that much of the N-terminus of the protein is predicted to be transmembrane,^4^ these deletions may have a profound impact on the structure of the protein. While our Western blot and proteomics data support N-terminal cleavage of the protein, it is possible that an intact N-terminus must be initially present to allow proper folding and insertion of RHBDL4 into the membrane prior to processing of the N-terminus.

Having observed that several mutants were labeled with the activity-based probe, we sought to determine whether they retained proteolytic activity. Based on a prior report that RHBDL4 cleaves amyloid precursor protein (APP),^11^ we investigated whether we could observe cleavage of APP by our hRHBDL4 mutants. We cotransfected cells with our desired hRHBDL4 construct and a construct^24^ encoding for APP fused to enhanced green fluorescent protein (EGFP) at its C-terminus (Figure 3D**)**. When wild-type hRHBDL4 and the APP-EGFP fusion were coexpressed, we observed several bands between 30–50 kDa using an in-gel fluorescence measurement that are not present in a control expressing APP-EGFP alone (Figure 3E). Cotransfection with the S144A mutant similarly resulted in only a single background cleavage band. Notably, the sizes of the cleavage products produced by wild-type hRHBDL4 are consistent with the previously observed C-terminal fragments^11,25^ fused to EGFP (∼27 kDa). We repeated the same experiment with our truncated mutants and observed a similar set of cleavage products for several mutants (Supplemental Figure 4). To account for the differences in APP-EGFP expression, we quantified the degree of APP cleavage by determining the ratio of the band densities for the fragments between 30–50 kDa with the full-length fusion protein (∼114 kDa). We found that the Δ278–315 and Δ261–315 mutants display comparable activity to wild-type hRHBDL4, consistent with the results of our ABPP gel (Figure 3F). The Δ243–315 and Δ219–315 mutants also retain proteolytic activity. These findings confirm the catalytic activity of truncated forms of hRHBDL4 and provide compelling evidence that our ABPP assay serves as an effective method for monitoring RHBDL4 activity.

Having established the ability of FP-Rh to detect active hRHBDL4, we then examined whether the ABPP assay could be used in a competitive format for inhibitor discovery. We tested an initial panel of compounds containing both commercial serine protease inhibitors (**MAFP**, **PMSF**, **AEBSF**, **TPCK**, **3,4-DCI**) and previously reported^17,27^ rhomboid protease inhibitors (**Bsc5195**, **WHP1A**, **WHP3A**) (Figure 4A, Supplemental Figure 5). Consistent with our previous work^17^ screening inhibitors against PARL and the bacterial rhomboid protease GlpG, we observed different levels of competition of probe labeling with this initial set of compounds (Figure 4B). While a second fluorophosphonate (**MAFP**) fully competes FP-Rh labeling, neither of the sulfonyl fluorides (**PMSF**, **AEBSF**) tested competes FP-Rh labeling. Both 3,4-dichloroisocoumarin (**3,4-DCI**) and a saccharin-based compound (**Bsc5195**), previously reported as a GlpG inhibitor, provide nearly complete competition of probe labeling of hRHBDL4 at 100 μM. However, **WHP3A**, which we previously observed to be an effective inhibitor in competitive ABPP assays with PARL,^17^ shows little-to-no inhibition of FP-Rh labeling of hRHBDL4. We observed a similar inhibition profile when we screened this panel of compounds against mRHBDL4 (Supplemental Figure 5). Using one of our initially discovered inhibitors (**BSc5195**), we also confirmed that competition of probe labeling was not accompanied by an alteration in the RHBDL4-associated bands in the Western blot (Supplemental Figure 6).

**Figure 4:**
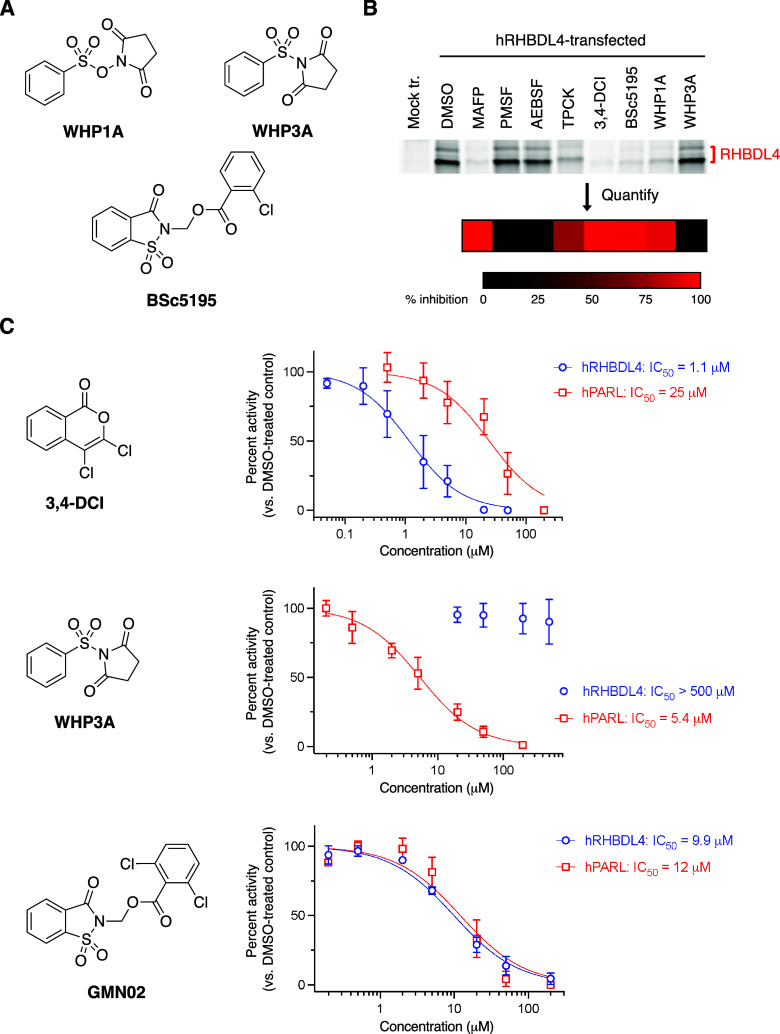
Competitive ABPP for the discovery of RHBDL4 inhibitors. (A) Structures of noncommercial compounds in panel; (B) Representative competitive ABPP gel for hRHBDL4-transfected HEK293T membrane proteome treated with 100 μM of each of the indicated compounds prior to FP-Rh treatment. Percent inhibition of labeling with each compound is presented in the heatmap with each value representing the average of *n* = 3 independent experiments. (C) Dose–response curves for each compound tested against hRHBDL4-transfected and hPARL-transfected HEK293T membrane proteomes. Data represent the mean ± standard deviation for *n* ≥ 3 independent experiments.

To explore the inhibitor preferences for each human rhomboid protease further, we determined IC_50_ values for two of our compounds against both hRHBDL4 and hPARL (Figure 4C, Supplemental Figure 7). We observed that **3,4-DCI** displays greater potency for hRHBDL4 than hPARL (IC_50_ values of 1.1 and 25 μM, respectively). Conversely, while the succinimide-containing **WHP3A** displays low micromolar potency for hPARL, it fails to inhibit hRHBDL4 at even high concentrations (up to 500 μM). Intrigued by the effectiveness of the saccharin-containing molecule **BSc5195** against multiple rhomboid proteases including GlpG,^27^ we synthesized a set of structural derivatives (**GMN01–07**) and investigated their ability to inhibit both hRHBDL4 and hPARL (Supplemental Figure 8). We found that all seven compounds similarly inhibit probe labeling of both hRHBDL4 and hPARL, suggesting that these saccharin-containing structures may have potential as broad-spectrum rhomboid protease inhibitors. Indeed, one of our newly synthesized saccharin structures, **GMN02**, is equipotent against both enzymes (Figure 4C). The differences observed with the isocoumarin, succinimide, and saccharin scaffolds are intriguing considering that they are proposed to be covalent inhibitors with related mechanisms of action. Though understanding the nature of these differences will be the subject of future work, these results provide encouraging precedent that selective inhibition among the human rhomboid proteases with small molecules is possible.

Despite the involvement of RHBDL4 in critical cellular processes, the development of chemical tools to study this enzyme has been limited to-date. We have demonstrated that fluorophosphonate reagents can be used as activity-based probes for RHBDL4 to empower further study of this enzyme. The use of an activity-based probe allowed us to visualize active forms of the enzyme that may have otherwise eluded detection. Our findings indicate that heterologously expressed RHBDL4 undergoes proteolytic processing at its C-terminus to generate multiple proteoforms that retain catalytic activity. We also obtained evidence that the enzyme undergoes processing at its N-terminus. A limitation of our study is that our experiments were conducted exclusively with an overexpression system; future work should investigate to what extent these events are observed for endogenous RHBDL4. These studies will likely require an activity-based probe with enhanced selectivity for RHBDL4. Our work strongly suggests that the cytosolic C-terminal domain is nonessential for RHBDL4’s enzymatic activity. Given that the C-terminal domain has been shown to mediate protein–protein interactions,^4,28,29^ future work should investigate how potential loss of these interacting motifs impacts pathways involving RHBDL4 and whether peptides cleaved from RHBDL4 might play signaling roles as has been proposed for PARL.^18^

We have also demonstrated that our ABPP assay provides a straightforward platform for RHBDL4 inhibitor discovery. With a small collection of compounds, we have already identified notable differences in the inhibitor preferences of RHBDL4 and PARL. Future work with expanded compound libraries should uncover inhibitors with enhanced potency that can selectively modulate the activities of individual human rhomboid proteases. Selective RHBDL4 inhibitors would represent valuable tools for probing the complex biology of this enzyme and could ultimately be used to investigate the therapeutic potential of RHBDL4 inhibition in diseases like cancer.

## Supporting Information Available

Experimental methods including protein expression, proteomics methods, and compound synthesis; supplementary data including figures and tables; compound characterization details (PDF)
