# Supplementary material for: Activity-Based Protein Profiling of RHBDL4 Reveals Proteolysis of the Enzyme and a Distinct Inhibitor Profile
Source: ACS Chem Biol. 2024 Jul 23;19(8):1674–82. doi: 10.1021/acschembio.4c00273 (PMC11334910; doi:10.1021/acschembio.4c00273)
Supplement: Supplementary file 1 — cb4c00273_si_001.pdf [file cb4c00273_si_001.pdf]

Supporting Information for

Activity-Based Protein Profiling of RHBDL4 Reveals Proteolysis of the Enzyme and a Distinct Inhibitor Profile

Cassondra C. Davies[1], Ren-Ming Hu[2], Paul J. Kamitsuka[1], Gabriel N. Morais[1], Regina Stasser de Gonzalez[1], Katelyn A. Bustin[1], Megan L. Matthews[2], William H. Parsons[1]

[1]Department of Chemistry and Biochemistry, Oberlin College, Oberlin, OH, USA
[2]Department of Chemistry, University of Pennsylvania, Philadelphia, PA, USA

Table of Contents

Supplementary Figures and Tables 3

Supplementary Methods 14

Supplementary References 24

NMR Spectra 25

Supplementary Figures and Tables

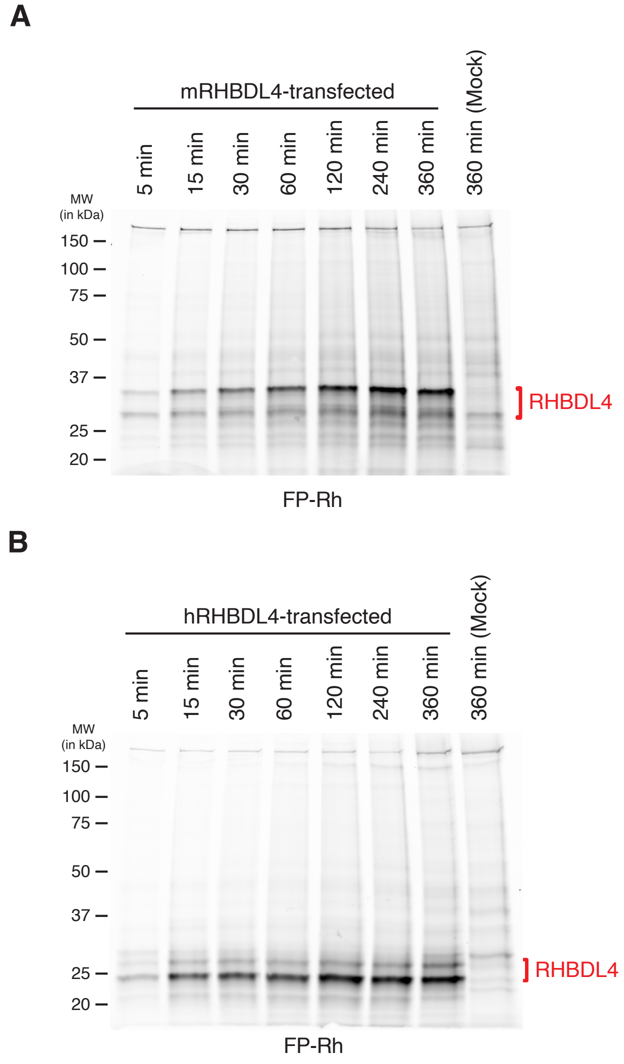

Supplemental Figure 1. FP-Rh labeling of RHBDL4 is time-dependent. (a) ABPP gel for mRHBDL4-transfected HEK293T membrane proteome treated with 1 μM FP-Rh for increasing amounts of time compared to a control transfected with empty vector ("Mock"). mRHBDL4 was expressed with N-terminal FLAG and myc tags. (b) ABPP gel for hRHBDL4-transfected HEK293T membrane proteome treated with 1 μM FP-Rh for increasing amounts of time compared to a control transfected with empty vector ("Mock"). hRHBDL4 was expressed with N-terminal FLAG and myc tags.

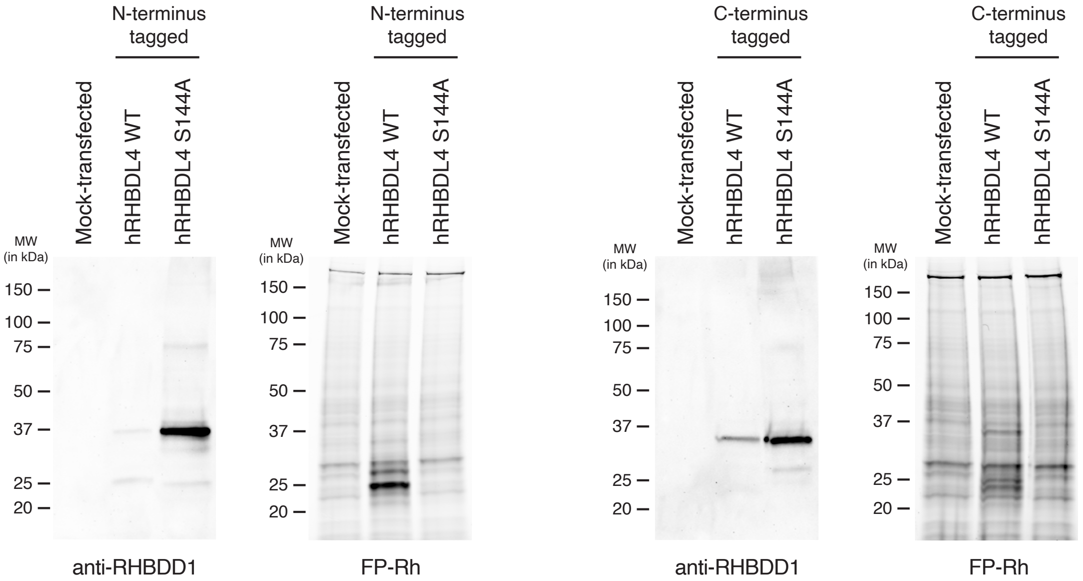

Supplemental Figure 2. Western blots and ABPP gels for membrane proteomes of HEK293T cells transfected with empty vector ("Mock"), wild-type human RHBDL4 (hRHBDL4), or the S144A mutant. Constructs encoding for either N-terminal epitope tags or C-terminal epitope tags were used as indicated. Proteomes were treated with 1 μM FP-Rh for 2 h.

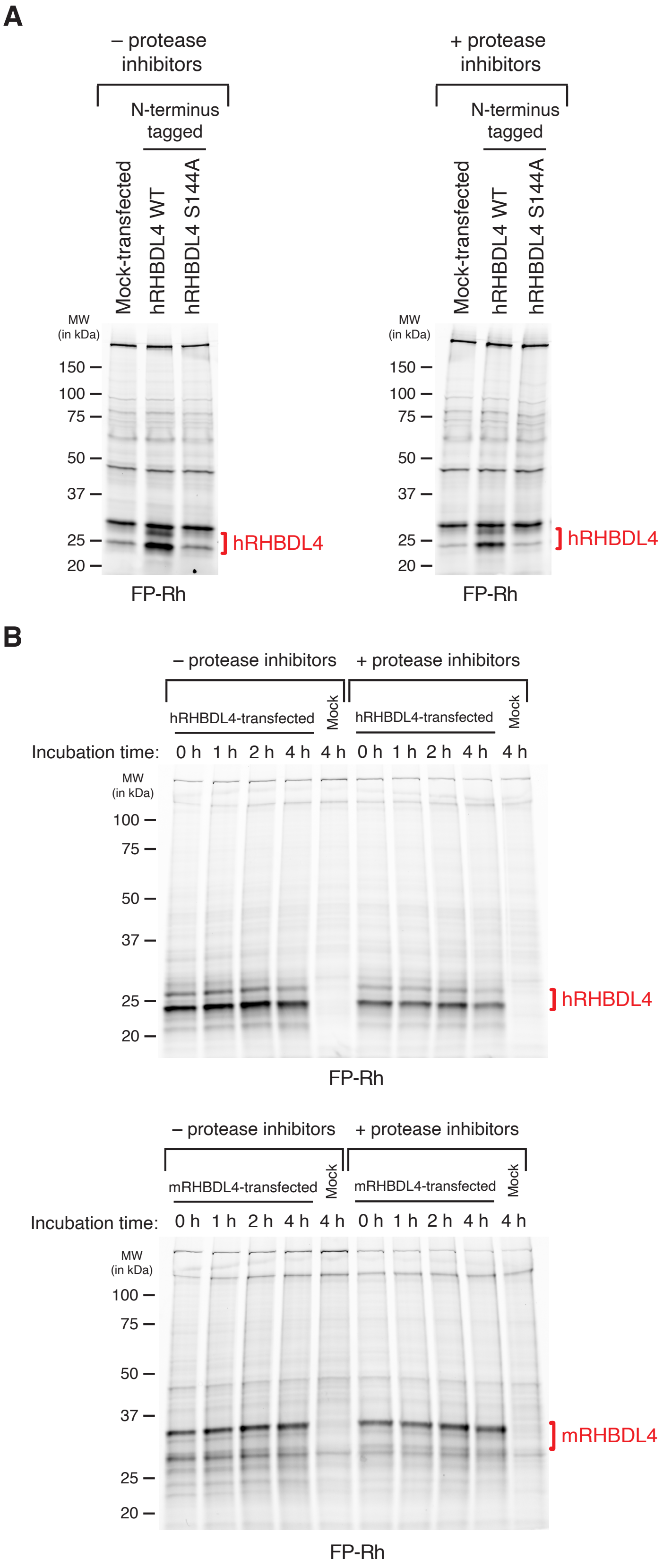
A
– protease inhibitors
N-terminus tagged
Mock-transfected
hRHBDL4 WT
hRHBDL4 S144A
MW (in kDa)
150
100
75
50
37
25
20
hRHBDL4
FP-Rh
+ protease inhibitors
N-terminus tagged
Mock-transfected
hRHBDL4 WT
hRHBDL4 S144A
hRHBDL4
FP-Rh
B
– protease inhibitors
+ protease inhibitors
hRHBDL4-transfected
Mock
Incubation time: 0 h 1 h 2 h 4 h 4 h 0 h 1 h 2 h 4 h 4 h
hRHBDL4
FP-Rh
mRHBDL4-transfected
mRHBDL4
FP-Rh

Supplemental Figure 3. The addition of protease inhibitors during lysis does not impact proteolysis of RHBDL4. (A) ABPP gels of the whole cell lysates of HEK293T cells transfected with empty vector, wild-type hRHBDL4, or the S144A mutant. Lysates were generated in the presence or absence of the cOmplete™ EDTA-free protease inhibitor cocktail (Roche) as indicated. (B) ABPP gels of the membrane proteomes of HEK293T cells transfected with empty vector, wild-type hRHBDL4, or wild-type mRHBDL4. Proteomes were generated in the presence or absence of the cOmplete™ EDTA-free protease inhibitor cocktail (Roche) as indicated. Proteomes were incubated at 37 °C for increasing amounts of time (from 0 to 4 hours) prior to treatment with 1 μM FP-Rh for 2 hours.

Sequence	Spectral counts N-terminal FLAG	Spectral counts C-terminal FLAG
L_{37}.NIWFFLNPQKPLYSSCLSVEK.C_{59}	1	
R_{6}.GINTGLILLLSQIFHVGINNIPPVTLATLALNIWFFLNPQKPLYSSCLSVEK.C_{59}	3	
W_{40}.FFLNPQKPLYSSCLSVEK.C_{59}	10	4
K_{58}.CYQQKDWQR.L_{68}	2	
R_{67}.LLLSPLHHADDWHLYFNMASMLWK.G_{92}	46	11
R_{98}.LGSRWFAYVITAFSVLTGVVYLLLQFAVAEFMDEPDFKR.S_{138}	2	
R_{137}.SCAVGFSGVLFALK.V_{152}	7	4
A_{149}.LKVLNNHYCPGGFVNILGFPVPNR.F_{174}	160	54
R_{173}.FACWVELVAIHLFSPGTSFAGHLAGILVGLMYTQGPLKK.I_{213}	5	3
K_{212}.IMEACAGGFSSSVGYPGRQ.Y_{232}	429	206
R_{230}.QYYFNSSGSSGYQDYYPHGRPDHYEEAPRN.Y_{261}	163	78
R_{259}.NYDTYTAGLSEEEQLERA.L_{278}	45	50
R_{276}.ALQASLWDR.G_{286}	4	4
R_{289}.NSPPPYGFHLSPEEMR.R_{306}	26	25
Total	***903***	***439***

Supplemental Table 1. Spectral counts for the tryptic peptides generated from enrichment of hRHBDL4 with FP-biotin from the membrane fractions of HEK293T cells transfected with either N-terminally or C-terminally epitope-tagged hRHBDL4.

Sequence	Spectral counts<br>Band 1	Band 2	Band 3
F_{20}.HVGINNIPPVTL.A_{33}	7	8	
W_{40}.FFLNPQKPL.Y_{50}	3	4	
L_{70}.SPLHHADDWHLY.F_{83}	3	3	3
F_{124}.AVAEFMDEPDFKRSCAVGF.S_{144}	2	2	4
L_{153}.NNHYCPGGF.V_{163}	1		
W_{177}.VELVAIHLF.S_{187}	1	1	
F_{192}.AGHLAGILVGL.M_{204}		1	
L_{180}.VAIHLFSPGTSF.A_{193}			1
L_{210}.KKIMEACAGGF.S_{222}	2	1	4
F_{221}.SSSVGYPGRQYY.F_{234}	3	4	1
Y_{242}.QDYYPHGRPDHYEEAPRNY.D_{262}	7	11	12
Y_{264}.TAGLSEEEQLERALQ.A_{280}	6	3	3
Total	***35***	***38***	***28***

Supplemental Table 2. Spectral counts for the chymotryptic peptides obtained for the primary bands (see **Figure 2** of main text) associated with hRHBDL4-transfected HEK293T membrane proteome.

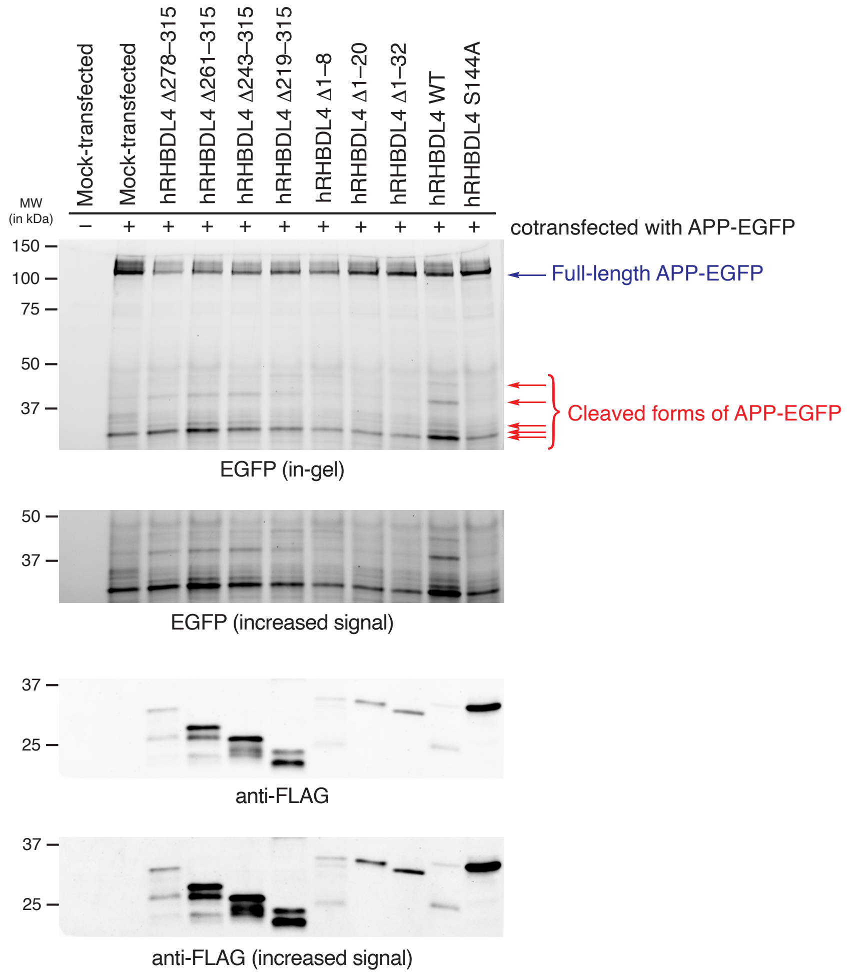

Supplemental Figure 4. Representative in-gel fluorescence image and Western blot of the whole cell lysates of HEK293T cells co-transfected as indicated. Arrows are used to indicate the positions of the full-length APP-EGFP fusion and EGFP fused to APP fragments. An anti-FLAG antibody was used to detect RHBDL4 expression.

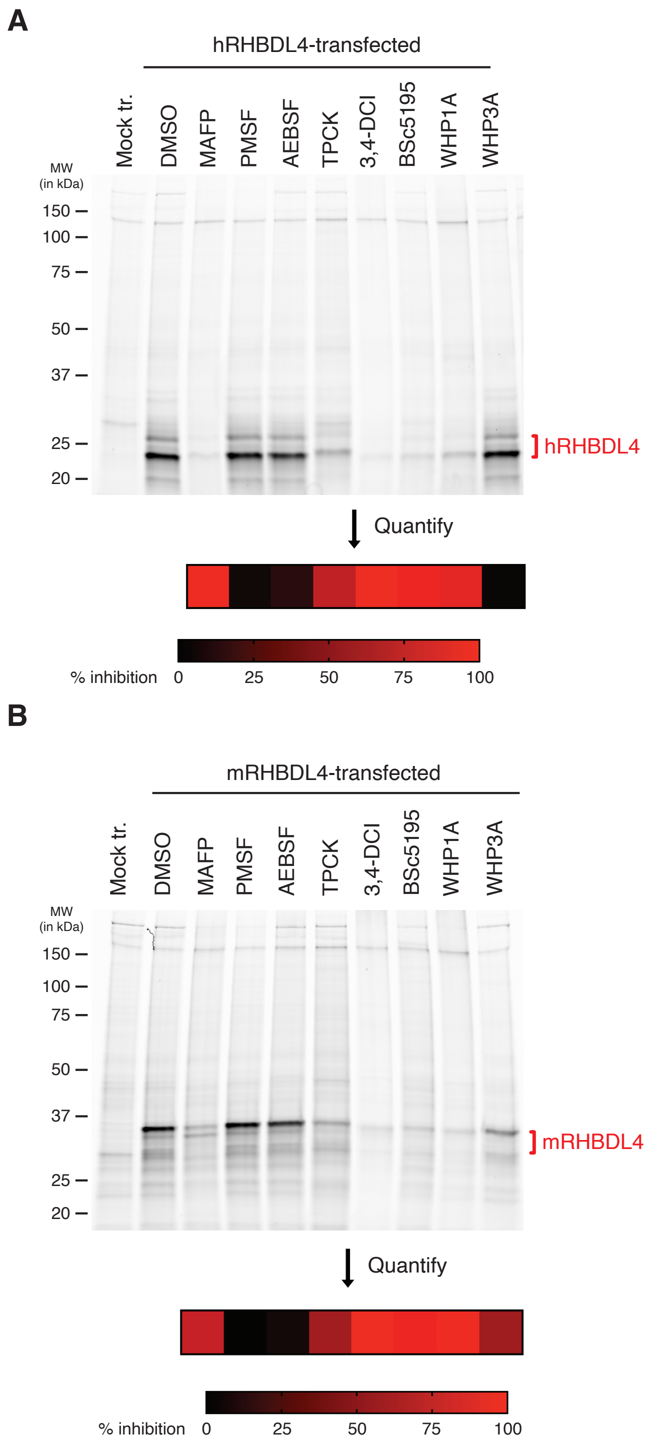

Supplemental Figure 5. Competitive ABPP with previously reported protease inhibitors. (A) Full gel image for cropped gel image presented in Figure 4B; (B) Representative competitive ABPP gel for mRHBDL4-transfected HEK293T membrane proteome treated with 100 μM of each of the indicated compounds prior to FP-Rh treatment. Percent inhibition of labeling with each compound is presented in the heatmap with each value representing the average of $n = 3$ independent experiments.

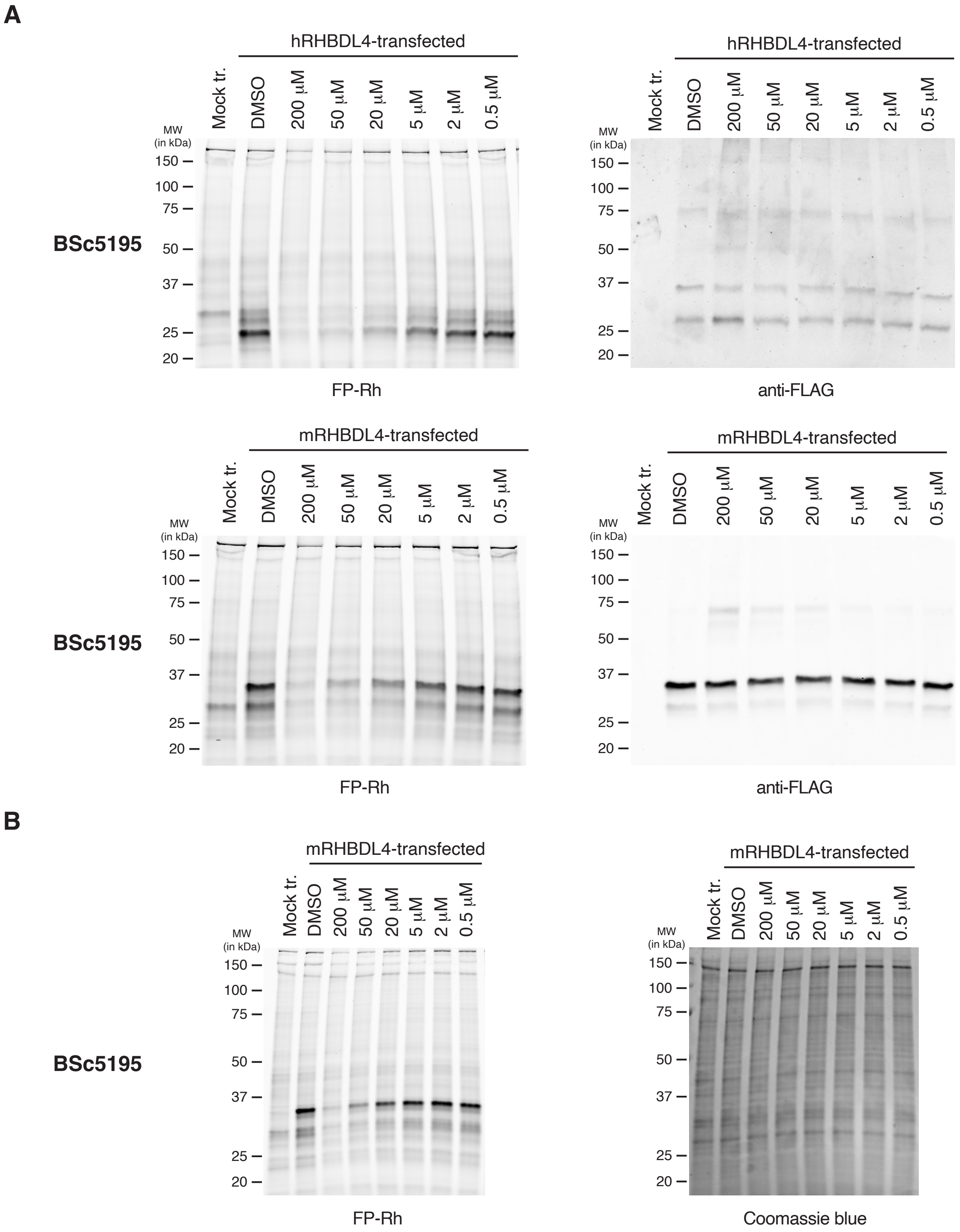

Supplemental Figure 6. Competition of FP-Rh labeling with BSc5915 does not lead to alterations in the Western blot or a Coomassie gel. (A) Representative ABPP gels and Western blots for hRHBDL4-transfected and mRHBDL4-transfected HEK293T membrane proteomes treated with the indicated concentrations of BSc5195 prior to FP-Rh treatment. (B) Representative ABPP gel and Coomassie-stained image for mRHBDL4-transfected HEK293T membrane proteome treated with the indicated concentrations of BSc5195 prior to FP-Rh treatment.

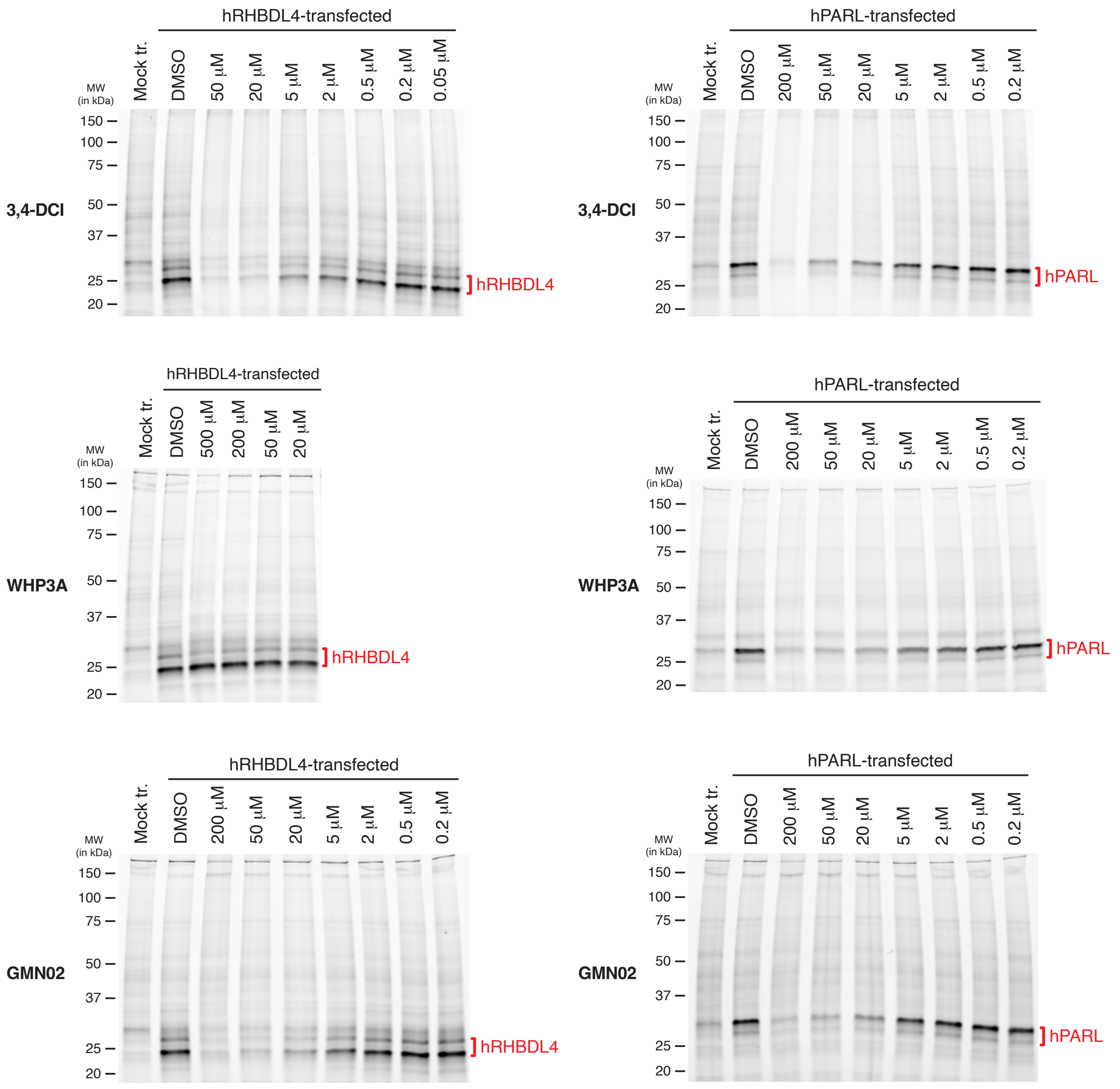

Supplemental Figure 7. Representative competitive ABPP gels for hRHBDL4-transfected and hPARL-transfected HEK293T membrane proteomes treated with the indicated concentrations of each compound prior to FP-Rh treatment.

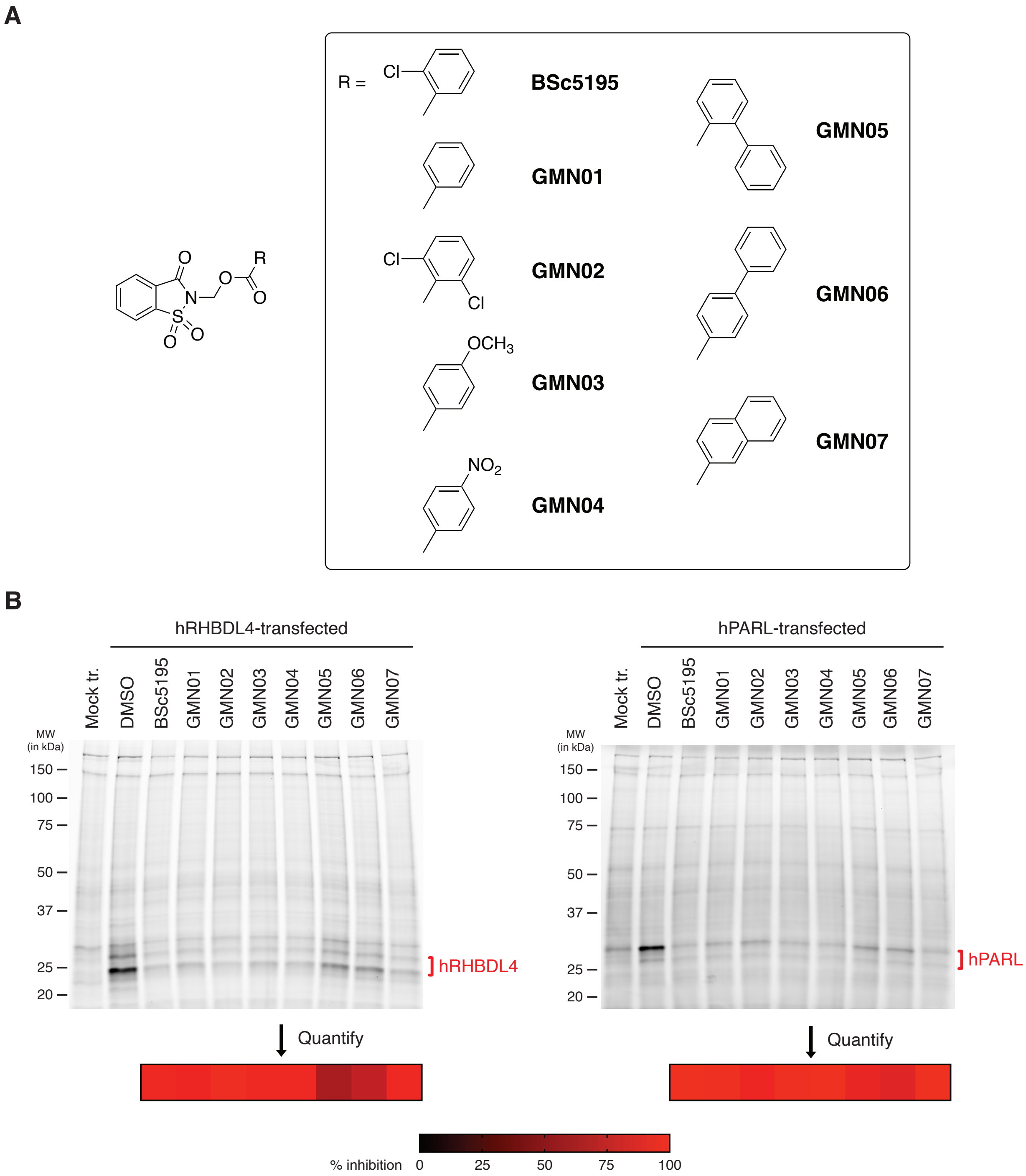

Supplemental Figure 8. A small library of saccharin compounds competes probe labeling of hRHBDL4 and hPARL. (A) Structures of the synthesized saccharin compounds; (B) Representative competitive ABPP gels for hRHBDL4 and hPARL-transfected HEK293T membrane proteomes treated with 100 μM of each of the indicated compounds prior to FP-Rh treatment. Percent inhibition of labeling with each compound is presented in the heatmap with each value representing the average of $n = 3$ independent experiments.

Supplementary Methods

DNA Construct Information. cDNA constructs encoding for mammalian expression of full-length wild-type human RHBDL4 (hRHBDL4) and full-length wild-type mouse RHBDL4 (mRHBDL4) with C-terminal FLAG and myc tags in the pCMV6-Entry vector were purchased from Origene (RC210708 and MR204515, respectively). cDNA constructs encoding for mammalian expression of full-length wild-type human RHBDL4 and wild-type mouse RHBDL4 with N-terminal FLAG and myc tags in the pCMV6-AN-Myc-DDK vector were generated by digestion with restriction enzymes (AsiSI and MluI) followed by ligation (Quick Ligation Kit, New England BioLabs) according to the manufacturer's protocol. pEGFP-n1-APP was a gift from Zita Balklava & Thomas Wassmer (Addgene plasmid #69924; http://n2t.net/addgene:69924; RRID: Addgene 69924). A cDNA construct encoding for mammalian expression of full-length wild-type human PARL with a C-terminal FLAG tag in the pcDNA3 vector was a gift from Luca Pellegrini (Addgene plasmid #13639; http://n2t.net/addgene:13639; RRID: Addgene 13639).

Site-Directed Mutagenesis. Mutations in hRHBDL4 and mRHBDL4 were generated according to the manufacturer's protocol for the Q5 Site-Directed Mutagenesis Kit (New England BioLabs). The following sets of primers were used:

**Primer name**	**Sequence**
hRHBDL4 S144A forward	5´-TGTAGGTTTCGCAGGAGTTTTGTTTG-3´
hRHBDL4 S144A reverse	5´-GCACAGCTCCTTTTGAAGTC-3´
mRHBDL4 S144A forward	5´-TGTGGGCTTCGCAGGAGTTTTGT-3´
mRHBDL4 S144A reverse	5´-GCACAGTTCCTTTTGAAGTC-3´
hRHBDL4 Δ1-8 forward	5´-AATACTGGACTTATTCTACTCC-3´
hRHBDL4 Δ1-20 forward	5´-CATGTTGGGATCAACAATATTC-3´
hRHBDL4 Δ1-32 forward	5´-GCAACTTTGGCCCTC-3´
hRHBDL4 Δ1-47 forward	5´-CCACTGTATAGCTCCTG-3´
hRHBDL4 Δ1-58 forward	5´-TGTTACCAGCAAAAAGAC-3´
hRHBDL4 N-term Δ reverse	5´-GGCGATCGCCTTATC-3´
hRHBDL4 C-term Δ forward	5´-ACGCGTTAAGCGGCC-3´
hRHBDL4 Δ278-315 reverse	5´-TGCTCTCTCGAGCTGTTC-3´
hRHBDL4 Δ261-315 reverse	5´-GTTCCTGGGTGCTTCTTC-3´
hRHBDL4 Δ243-315 reverse	5´-ATATCCAGAGCTGCCTGAAC-3´
hRHBDL4 Δ219-315 reverse	5´-TGCACATGCTTCCATGATTTTC-3´

All DNA sequencing was performed by Eurofins Genomics.

Mammalian Expression of RHBDL4, PARL, and EGFP-fused APP695. HEK293T cells (ATCC CRL-3216) were cultured in DMEM (ATCC), supplemented with 10% (v/v) fetal bovine serum (ATCC) and penicillin-streptomycin (GE Life Sciences) at 37 °C and 5% CO_2. For recombinant expression, HEK293T cells were grown to 50% confluence in a 10 cm tissue culture plate and transiently transfected with 4 μg of the desired construct using polyethyleneimine 'MAX' (MW 40,000, PEI; Polysciences, Inc.) as the transfection reagent per the manufacturer's protocol. Alternatively, HEK293T cells were grown to 50% confluence in a 6-well plate, and each well was transiently transfected with 1 μg of the desired construct using polyethyleneimine 'MAX' as the transfection reagent per the manufacturer's protocol. 'Mock' transfected cells were transfected

with 4 μg or 1 μg of empty vector, respectively. For co-transfection experiments, HEK293T cells were grown to 50% confluence in a 6-well plate and transiently transfected with 1 μg of the desired RHBDL4 construct and 0.5 μg of the pEGFP-n1-APP construct. Cells were incubated at 37 °C for 24 hours after transfection, at which point the media was changed. 48 hours after transfection, cells were washed with Dulbecco's phosphate-buffered saline (PBS, Corning) and harvested by scraping.

Mammalian Cell Lysate Preparation. HEK293T cell pellets were suspended in either 500 μL (if harvested from a 10 cm plate) or 200 μL (if harvested from a well of a 6-well plate) of ice-cold PBS, lysed by sonication at 4 °C, and centrifuged at 1400*g* for 3 min to remove cell debris and generate the whole cell lysate. The membrane and soluble fractions were separated by ultra-centrifugation at 100,000*g* for 45 minutes at 4 °C. After isolating the soluble fraction as the supernatant, the membrane fraction pellet was washed with PBS (x3) and then resuspended in PBS by sonication. The resuspended membrane pellet is referred to as the membrane proteome. Protein concentrations were determined using the DC Protein Assay Kit II (Bio-Rad) according to the manufacturer's protocol.

Gel-based ABPP analysis for *in vitro* treatment. Cell proteomes were diluted to 1 mg mL^{-1} in PBS. Each proteome sample (30 μL) was treated with 0.6 μL of FP-rhodamine (FP-Rh, 1 μM) for 2 h at 37 °C. The reactions were then quenched by addition of 4X SDS-PAGE loading buffer (10 μL). Proteomes were analyzed using 10% TGX™ gels (Bio-Rad). Samples were visualized in-gel using a ChemiDoc™ MP Imaging System (Bio-Rad). The fluorescence from rhodamine is presented in grayscale. 30-90 s exposure times were used for RHBDL4 and PARL-expressing proteomes. Relative band intensities were quantified using ImageJ software (http://imagej.nih.gov/ij/).

Competitive gel-based ABPP analysis for inhibitor treatment. Competitive gel-based ABPP experiments were performed as previously described[1]. Stock solutions (either 10 or 100 mM) were generated of each compound in DMSO. A series of dilutions ranging from 2.5 μM to 2.5 mM were then generated from each stock solution. Cell proteomes (30 μL, 1 mg mL^{-1}) were treated with 0.6 μL of the appropriate inhibitor solution (e.g., 0.6 μL of a 2.5 mM inhibitor solution to achieve a final concentration of 500 μM) or DMSO alone (as a control) for 30 min at 37 °C. Proteomes were then treated with 0.6 μL of FP-rhodamine (FP-Rh, 1 μM) for 2 h at 37 °C. The reactions were then quenched by addition of 4X SDS-PAGE loading buffer (10 μL) and analyzed as described above. Concentration-dependence inhibition curves obtained from three or more trials at each inhibitor concentration were fit using GraphPad Prism 10 (for Mac OS) software to obtain IC_{50} values.

Western blotting. Cell proteomes were separated by SDS-PAGE, transferred to nitrocellulose membrane (60 V for 90 min), and blocked with 5% milk in TBS-Tween. The following primary antibodies were used at the indicated dilutions: anti-FLAG (Rabbit, Sigma, F7425, 1:400) and anti-RHBDD1 (Rabbit, Sigma, HPA013972, 1:400). A peroxidase-labeled anti-rabbit secondary antibody (Cytiva Life Sciences, 1:10,000) was used for visualization. The membrane was then imaged using Amersham ECL Western blotting detection reagents (Cytiva) according to the manufacturer's protocol and a ChemiDoc™ MP Imaging System (Bio-Rad).

Gel-based analysis of APP695 cleavage. Proteomes of cells expressing the APP695-EGFP fusion (30 μL, 1 mg mL^{-1}) were prepared and mixed with SDS-PAGE loading buffer (10 μL).

Proteomes were analyzed using 10% TGX™ gels (Bio-Rad). Samples were visualized in-gel using a ChemiDoc™ MP Imaging System (Bio-Rad). The fluorescence from EGFP is presented in grayscale. 30-90 s exposure times were used for APP-EGFP-expressing proteomes. Relative band intensities were quantified using ImageJ software (http://imagej.nih.gov/ij/).

Data analysis. Analyses, including generation of heatmaps and IC_{50} determination, were performed using the GraphPad Prism 10 (for Mac OS) software. Data derived from three or more replicates are shown as mean values ± s.d.

Proteome preparation of cell lysates for in vitro gel- and MS-based experiments. Cell pellets were resuspended in ice-cold PBS (pellet/PBS = 1/60, *v/v*), and lysed (3 x 10 pulses, 0.3 seconds on, 2 seconds off, 15% energy) by a sonicator (Branson SFX250 Sonifier) equipped with a 102C microtip. Soluble and membrane fractions were separated by ultracentrifugation (Thermofisher S55-A2 rotor; 100 kg, 30 min, 4°C). The soluble fraction was removed; the membrane fraction was gently washed 3 times with PBS, then resuspended in 300 μL PBS followed by probe sonication. The concentration of each membrane fraction was determined by the DC protein assay kit (Bio-Rad) on a microplate reader (Biotek Elx808 plate reader).

FP-biotin enrichment. Membrane fractions of hRHBDL4-transfected HEK293T cells were diluted to 1 mg mL^{-1}. To each sample (1 mL), 10 μL of 10 mM FP-biotin was added. Upon addition of the probe solution, each sample was immediately vortexed and then allowed to react at room temperature under rotation for 2 h. Sequential addition of a mixture with pre-chilled methanol (MeOH, 2 mL), chloroform ($CHCl_3$, 0.5 mL) and PBS (1 mL) on ice quenched the reaction. The precipitated proteome was centrifuged (5,000 g, 10 min, 4 °C) to fractionate the protein interphase from the organic and aqueous solvent layers. The protein pellet was washed with cold 1:1 MeOH:$CHCl_3$ (3 × 1 mL), mildly sonicated in cold 4:1 MeOH:$CHCl_3$ (2.5 mL) and pelleted once more by centrifugation (5,000 g, 10 min, 4 °C). The remaining protein precipitate was redissolved by mild sonication in a freshly prepared solution of proteomics-grade urea (500 μL, 6 M in PBS). Disulfides were reduced with TCEP (9 mM) pre-neutralized with potassium carbonate (27 mM) for 30 min at 37 °C. Reduced thiols were then alkylated by iodoacetamide (45 mM) for 30 min at ambient temperature protected from light. SDS [2% (w/v)] was added to ensure complete denaturation. The solution was diluted to 0.2% SDS with PBS (~5 mL) and incubated with pre-equilibrated streptavidin agarose resin (50 μL column volume, 100 μL 1:1 slurry, Pierce) for ~1.5–2 h at ambient temperature on a rotator. The streptavidin beads were collected by centrifugation (1,400 g, 1–2 min) and sequentially washed with 0.2% SDS in PBS (3 × ~10 mL), detergent-free PBS (3 × ~10 mL) and H_2O (3 × ~10 mL) to remove unbound protein, excess detergent, and small molecules. The resin was transferred to a Protein LoBind tube (Eppendorf or BioPioneer), and bound proteins were digested on-bead overnight at 37 °C in ~200 μL total volume containing sequencing grade porcine trypsin (2 μg, Promega) in the presence of urea (2 M in PBS) and $CaCl_2$ (1mM). The proteolyzed supernatant was transferred to a fresh Protein Lobind tube, acidified with formic acid (5%) to inactivate trypsin, and stored at -80 °C until analyzed.

Gel-based analysis of probe-labeled proteins. Membrane fractions were diluted to 1 mg mL^{-1}. To each sample (90 μL), 1.8 μL of 50 μM FP-Rh was added. Upon addition of the probe solution, each sample was immediately vortexed and then allowed to react at room temperature under rotation for 2 h before being quenched by addition of 30 μL of 4X sodium dodecyl sulfate (SDS) loading buffer. Samples (20 μL/ well for 28-well gel) were resolved by SDS-PAGE (10%

acrylamide self-made long gel) and visualized by in-gel fluorescence scanning on a ChemiDoc MP Imaging System (Bio-Rad).

In-gel digestion. For in-gel digestion sample preparation, membrane fractions were diluted to 3 mg mL^{-1}and split into two groups. The first group (90 μL each) was treated with 1.8 μL of 50 μM FP-Rh for 2 h before adding 30 μL of 4X SDS loading buffer; these samples were designated as the labeled proteomes. The second group (300 μL each) was directly mixed with 100 μL of 4X SDS loading buffer; these samples were designated as the unlabeled proteome. One labeled proteome sample (40 μL) was resolved by SDS-PAGE (10% acrylamide self-made long gel) next to 4 parallel unlabeled proteome samples. The gel was visualized by in-gel fluorescence scanning on a ChemiDoc MP Imaging System (Bio-Rad). The actual size of gel was printed out and put underneath the gel with a glass plate in between. The molecular weight ladder was used to align the gel and the print-out.

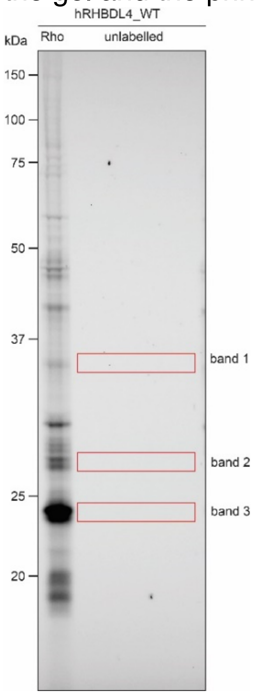

The full-length band at around 37 kDa and two cleaved bands at around 25 kDa on the SDS-PAGE gel were manually excised and chopped into small pieces. The gel pieces were washed with 100 mM ammonium bicarbonate (2 x 0.5 mL) followed by dehydration with acetonitrile (2 x 0.5 mL) until the gel pieces were completely opaque. Cysteines were reduced by rehydration in TCEP (10 mM in 100 mM ammonium bicarbonate) for 30 min at 37 ^{o}C. The gel pieces were dehydrated again using 0.5 mL acetonitrile for 10 min followed by alkylation with iodoacetamide (55 mM in 100 mM ammonium bicarbonate) for another 30 min at ambient temperature protected from light. Then the gel pieces were dehydrated again with acetonitrile. After removal of solution, the gel pieces were dried under vacuum. A solution of trypsin (4.8 μg trypsin in 480 μL PBS) or chymotrypsin (4.8 μg chymotrypsin in 480 μL PBS) was added to the gel pieces, which were incubated at 37 ^{o}C (25 ^{o}C for chymotrypsin) overnight. The next day, the supernatant was transferred into a new tube. A volume of 500 μL elution buffer (1:1 PBS/acetonitrile + 5% formic acid) was added to the gel pieces, and the samples were incubated for 30 min at room temperature. The second supernatant was then removed and combined with the first supernatant. The combined solution was put under vacuum to reduce the volume and then followed by the desalting protocol.

Peptide desalting and data analysis. Peptide samples were desalted prior to analysis by using in-house packed stage-tips. Stage-tips were manufactured by sealing five disks of C18 material (cat. No.: 2315, Empore, 3M Company) at the bottom of a P200 tip. C18 disks were cut by sample corers (cat. No.: 18035-02, Fine Science Tools). Stage-tips were equilibrated with 50 μL of methanol, 50 μL of 80% acetonitrile in H_2O containing 0.1% formic acid (FA), and 50 μL of water containing 0.1% FA by centrifugation (1,000 g, ~1-2 min). The sample was loaded to the stage-tip and centrifuged to flow through, washed with 75 μL of water containing 0.1% FA. The stage-tip was transferred to a new collection tube, eluted by 75 μL of 80% acetonitrile in H_2O containing 0.1% FA. The sample was dried by SpeedVac (SAVANT SVC100H Refrigerated Condensation Trap) under vacuum for 30-60 min at room temperature, stored at –80 °C. Prior to analysis, the sample was dissolved in 10 μL of 2% acetonitrile in H_2O containing 0.1% FA and transferred to an LC vial. Unless otherwise noted, an aliquot (5 μL) was injected into LC-MS/MS system.

Peptide identification. Methods for sample analysis using LC-MS/MS and peptide identification were adapted from previous studies.[2] From each of the five .raw files (one for each salt 'bump')

generated by the instrument (Xcalibur software), the MS2 spectra for all fragmented parent ions (.ms2 file) were extracted using RawConverter18 with monoisotopic selection (2015 released; http://fields.scripps.edu/rawconv). Each .ms2 file was searched using the ProLuCID algorithm against a reverse-concatenated, nonredundant database of the human proteome (Uniprot release –11/05/2012) and filtered using DTASelect 2.0 within the Integrated Proteomics Pipeline (IP2) software. Cysteine residues were searched with a static modification for S-carbamidomethylation (+57.02146). Methionine residues were searched with up to one differential modification for oxidation (+15.9949 Da). Peptides were required to have at least one tryptic terminus but an unlimited number of missed cleavages were allowed in the database search. The parent ion mass tolerance for a minimum envelope of three isotopic peaks was set to 50 ppm, the minimum peptide length was six residues, the false-positive rate was set at 2% or lower and at least two peptides of a protein must be detected.

De novo prediction of proteolytic processing cleavage site to generate the active enzyme. An Excel file containing the information of spectral counts for each of the corresponding RHBDL4 protein fragments (band 1, band 2, and band 3) identified in each cut band was extracted from ip2. Spectral counts for the same peptide fragments with different charge states were summed. All three sets of data were merged into one for comparison.

Synthetic chemistry

Chemical reagents. FP-Rh (ActivX™ TAMRA-FP) was purchased from Thermo Fisher Scientific. MAFP, PMSF, AEBSF, TPCK, and 3,4-DCI were purchased from Cayman Chemicals. WHP1A and WHP3A were synthesized as described previously.[3] All other compounds were synthesized in-house by procedures described below using starting materials and solvents commercially obtained from Sigma-Aldrich, VWR, Combi-Blocks, and Cambridge Isotope Laboratories. Product purification was accomplished through forced flow chromatography on Silicycle ultrapure silica gel (40-63 μm). Thin layer chromatography was performed on SiliaPlate silica gel F254 plates (250 μm). Visualization of the developed plate was accomplished by fluorescence quenching and by staining with either aqueous potassium permanganate ($KMnO_4$) or ceric ammonium molybdate (CAM) solution.

Complete characterization data for synthesized compounds is provided below. Nuclear magnetic resonance spectra were acquired on a Varian spectrometer operating at 400 MHz and 100 MHz for ^{1}H and ^{13}C, respectively. Spectra are referenced internally according to residual solvent signals. Data for ^{1}H NMR are recorded as follows: chemical shift (δ, ppm), multiplicity (s, singlet; d, doublet; t, triplet; q, quartet; quint, quintet; m, multiplet; br, broad), integration, coupling constant (Hz). Data for ^{13}C NMR are reported in terms of chemical shift (δ, ppm). Melting points were collected using a Thomas Hoover Uni-melt Capillary Melting Point Apparatus. High-resolution mass spectra for compounds previously unreported in the literature were obtained from the mass spectrometry facility in the Department of Chemistry at the University of Michigan using an Agilent Q-TOF HPLC-MS instrument.

All saccharin-containing structures were synthesized from a common intermediate **I**. **General Method A** was then used to convert intermediate **I** to each of the structures.

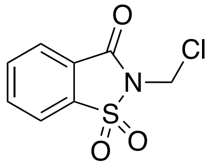

Intermediate **I**

Intermediate **I** was synthesized according to previously reported methods.[4,5] Briefly, saccharin (1.83 g, 10.0 mmol, 1.0 eq.) and formaldehyde (2.4 mL of a 37 % solution in water, 30.0 mmol, 3.0 eq.) were placed in 10 mL of water and stirred at 100 °C for 4.5 h. The reaction mixture was then cooled to 0 °C, and the resulting white precipitate (1.73 g, 8.1 mmol) was collected by filtration, washed with cold water, and used in the next step without further purification. The intermediate (1.42 g, 6.7 mmol, 1.0 eq.) was placed in neat thionyl chloride (1.45 mL, 20.0 mmol, 3.0 eq.), and the resulting mixture was stirred at 77 °C for 1.5 h. The mixture was concentrated under reduced pressure and then purified by column chromatography (70 % DCM/hexanes) to give N-chloromethyl saccharin (intermediate I) (1.03 g, 4.5 mmol, 54 % over two steps).

General Method A

To a solution of 1.0 eq. of intermediate **I** in dry acetonitrile (to 0.2 M) was added 2.0 eq. of the desired carboxylic acid and 2.0 eq. of triethylamine. The reaction mixture was stirred at 82 °C for

7.5-25 h and then cooled to 23 °C. The mixture was diluted in DCM and transferred to a separatory funnel along with water. The layers were separated, and the organic layer was washed with saturated sodium bicarbonate solution. The organic layer was then dried over Na_2SO_4, filtered, and concentrated. The product was purified by column chromatography and/or trituration with methanol.

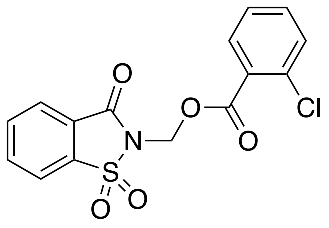

BSc5195

BSc5195 was synthesized by General Method A and purified by column chromatography (2 % acetone/DCM) and trituration in MeOH to give a white solid (257 mg, 84 % yield).

^{1}H-NMR (400 MHz, $CDCl_3$) δ 8.14 (d, *J* = 8.1 Hz, 1H), 8.00 – 7.85 (m, 4H), 7.48 – 7.40 (m, 2H), 7.33 – 7.27 (m, 1H), 6.09 (s, 2H) ppm.
^{13}C-NMR (100 MHz, $CDCl_3$) δ 163.9, 158.2, 137.6, 135.7, 134.7, 134.4, 133.4, 132.0, 131.2, 128.2, 126.7, 126.4, 125.8, 121.3, 61.7 ppm.
m.p. 119-121 ºC.

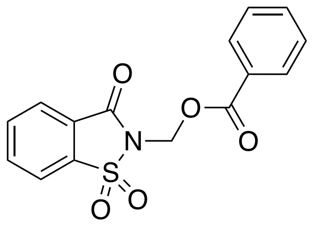

GNM01

Compound GNM01 was synthesized by General Method A and purified by trituration in MeOH to give a white solid (90.9 mg. 69 % yield).

^{1}H-NMR (400 MHz, $CDCl_3$) δ 8.11 (d, *J* = 6.8 Hz, 1H), 8.06 (dd, *J* = 8.3, 1.4 Hz, 2H), 7.98 – 7.82 (m, 3H), 7.56 (t, *J* = 7.4 Hz, 1H), 7.42 (t, *J* = 7.8 Hz, 2H), 6.09 (s, 2H) ppm.
^{13}C-NMR (100 MHz, $CDCl_3$) δ 165.3, 158.3, 137.8, 135.7, 134.7, 133.7, 130.1, 128.8, 128.5, 126.5, 125.8, 121.3, 61.6 ppm.
m.p. 108-110 ºC.

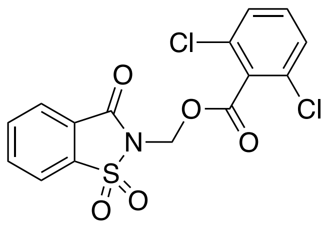

GMN02

Compound GNM02 was synthesized by General Method A and purified by trituration in MeOH to give a white solid (72.5 mg, 45 % yield).

^{1}H-NMR (400 MHz, $CDCl_3$) δ 8.13 (d, *J* = 7.6 Hz, 1H), 8.00 – 7.85 (m, 3H), 7.34 – 7.24 (m, 3H, signal partially obscured due to overlapping NMR solvent signal), 6.09 (s, 2H) ppm.
^{13}C-NMR (100 MHz, $CDCl_3$) δ 163.6, 158.3, 137.9, 135.8, 134.8, 132.4, 132.2, 131.5, 128.1, 126.5, 126.0, 121.5, 62.2 ppm.
m.p. 155-157 ºC.
HRMS (ES^+) calculated for $C_{15}H_9Cl_2NO_5S$ 384.9578; found 385.9645 (MH^+).

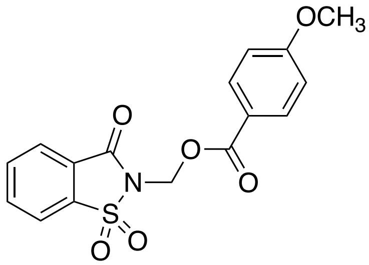

GNM03

Compound GNM03 was synthesized by General Method A and purified by trituration in MeOH to give a white solid (64.8 mg, 44 % yield).

^{1}H-NMR (400 MHz, $CDCl_3$) δ 8.13 (d, *J* = 6.9 Hz, 1H), 8.03 (d, *J* = 8.9 Hz, 2H), 8.00 – 7.83 (m, 3H), 6.90 (d, *J* = 8.9 Hz, 2H), 6.07 (s, 2H), 3.85 (s, 3H) ppm.
^{13}C-NMR (100 MHz, $CDCl_3$) δ 165.0, 164.1, 158.4, 138.0, 135.6, 134.7, 132.4, 126.8, 125.9, 121.4, 121.3, 113.9, 61.5, 55.6 ppm.
m.p. 138-139 ºC.

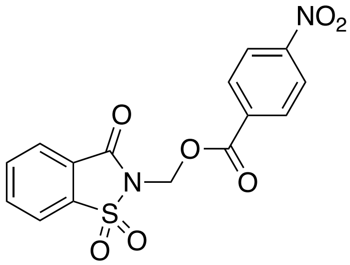

GNM04

Compound GNM04 was synthesized by General Method A and purified by column chromatography (1 % acetone/DCM) and trituration in MeOH to give a light-yellow solid (151 mg, 92 % yield).

^{1}H-NMR (400 MHz, $CDCl_3$) δ 8.30 – 8.19 (m, 4H), 8.14 (d, *J* = 6.8 Hz, 1H), 8.02 – 7.86 (m, 3H), 6.12 (s, 2H) ppm.
^{13}C-NMR (100 MHz, $CDCl_3$) δ 163.5, 158.2, 151.0, 137.7, 135.9, 134.9, 134.2, 131.3, 126.5, 126.0, 123.7, 121.4, 62.1 ppm.
m.p. 160-161 ºC.

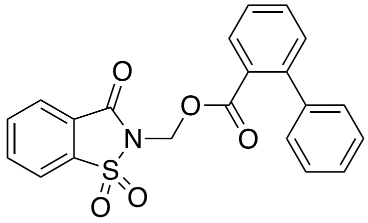

GNM05

Compound GNM05 was synthesized by General Method A and purified by column chromatography (30 % ethyl acetate/hexanes) and trituration in MeOH to give a white solid (138 mg, 82 % yield).

^{1}H-NMR (400 MHz, $CDCl_3$) δ 8.06 (d, *J* = 7.2 Hz, 1H), 7.96 – 7.81 (m, 4H), 7.52 (td, *J* = 7.5, 1.5 Hz, 1H), 7.39 (td, *J* = 7.6, 1.3 Hz, 1H), 7.35 – 7.27 (m, 3H), 7.24 (t, *J* = 7.7 Hz, 2H), 7.12 (t, *J* = 7.3 Hz, 1H), 5.85 (s, 2H) ppm.
^{13}C-NMR (100 MHz, $CDCl_3$) δ 166.8, 158.0, 143.3, 140.9, 137.7, 135.6, 134.6, 132.0, 131.1, 130.6, 129.0, 128.6, 128.0, 127.3, 127.2, 126.7, 125.7, 121.3, 60.9 ppm.
m.p. 102-103 ºC.
HRMS (ES^+) calculated for $C_{21}H_{15}NO_5S$ 393.0671; found 394.0739 (MH^+).

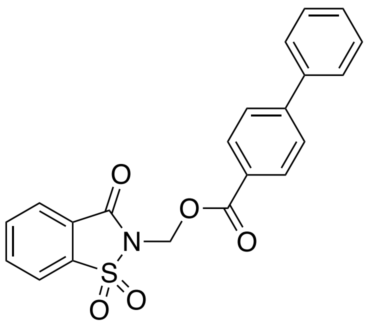

GNM06

Compound GNM06 was synthesized by General Method A and purified by column chromatography (2 % acetone/DCM) and trituration in MeOH to give a white solid (124 mg, 73 % yield).

^{1}H-NMR (400 MHz, $CDCl_3$) δ 8.14 (d, *J* = 8.5 Hz, 3H), 8.00 – 7.84 (m, 3H), 7.65 (d, *J* = 8.6 Hz, 2H), 7.60 (d, *J* = 6.9 Hz, 2H), 7.46 (t, *J* = 7.4 Hz, 2H), 7.39 (t, *J* = 7.3 Hz, 1H), 6.12 (s, 2H) ppm.
^{13}C-NMR (100 MHz, $CDCl_3$) δ 165.2, 158.3, 146.5, 139.9, 137.9, 135.7, 134.7, 130.7, 129.1, 128.4, 127.6, 127.4, 127.3, 126.7, 125.9, 121.4, 61.6 ppm.
m.p. 181-182 °C.
HRMS (ES^+) calculated for $C_{21}H_{15}NO_5S$ 393.0671; found 394.0740 (MH^+)

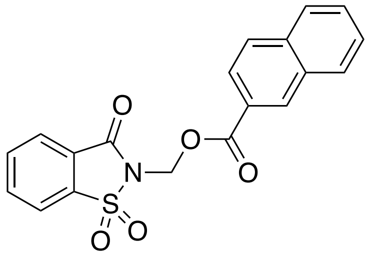

GNM07

Compound GNM07 was synthesized by General Method A and purified by column chromatography (1 % acetone/DCM) and trituration in MeOH to give an off-white solid (97.5 mg, 61 % yield).

^{1}H-NMR (400 MHz, $CDCl_3$) δ 8.65 (s, 1H), 8.15 (d, *J* = 6.9 Hz, 1H), 8.08 (dd, *J* = 8.7, 1.8 Hz, 1H), 8.00 – 7.84 (m, 6H), 7.59 (td, *J* = 7.6, 1.4 Hz, 1H), 7.53 (td, *J* = 7.5, 1.4 Hz, 1H), 6.17 (s, 2H) ppm.
^{13}C-NMR (100 MHz, $CDCl_3$) δ 165.5, 158.4, 137.8, 135.9, 135.6, 134.7, 132.4, 132.0, 129.6, 128.7, 128.4, 127.9, 126.8, 126.6, 126.1, 125.9, 125.3, 121.3, 61.7 ppm.
m.p. 152-153 °C.

References

(1) Li, W., Blankman, J. L., and Cravatt, B. F. (2007) A functional proteomic strategy to discover inhibitors for uncharacterized hydrolases. *J Am Chem Soc 129*, 9594–9595.

(2) Lin, Z., Wang, X., Bustin, K. A., Shishikura, K., McKnight, N. R., He, L., Suciu, R. M., Hu, K., Han, X., Ahmadi, M., Olson, E. J., Parsons, W. H., and Matthews, M. L. (2021) Activity-Based Hydrazine Probes for Protein Profiling of Electrophilic Functionality in Therapeutic Targets. *ACS Cent Sci 7*, 1524–1534.

(3) Parsons, W. H., Rutland, N. T., Crainic, J. A., Cardozo, J. M., Chow, A. S., Andrews, C. L., and Sheehan, B. K. (2021) Development of succinimide-based inhibitors for the mitochondrial rhomboid protease PARL. *Bioorg Med Chem Lett 49*, 128290.

(4) Subramanyam, C., Bell, M. R., Carabateas, P., Court, J. J., Dority Jr., J. A., Ferguson, E., Gordon, R., Hlasta, D. J., Kumar, V., and Saindane, M. (1994) 2,6-Disubstituted Aryl Carboxylic Acids, Leaving Groups "Par Excellence" for Benzisothiazolone Inhibitors of Human Leukocyte Elastase. *J Med Chem 37*, 2623–2626.

(5) Goel, P., Jumpertz, T., Mikles, D. C., Tichá, A., Nguyen, M. T. N., Verhelst, S., Hubalek, M., Johnson, D. C., Bachovchin, D. A., Ogorek, I., Pietrzik, C. U., Strisovsky, K., Schmidt, B., and Weggen, S. (2017) Discovery and Biological Evaluation of Potent and Selective N-Methylene Saccharin-Derived Inhibitors for Rhomboid Intramembrane Proteases. *Biochemistry 56*, 6713–6725.

BSc5195

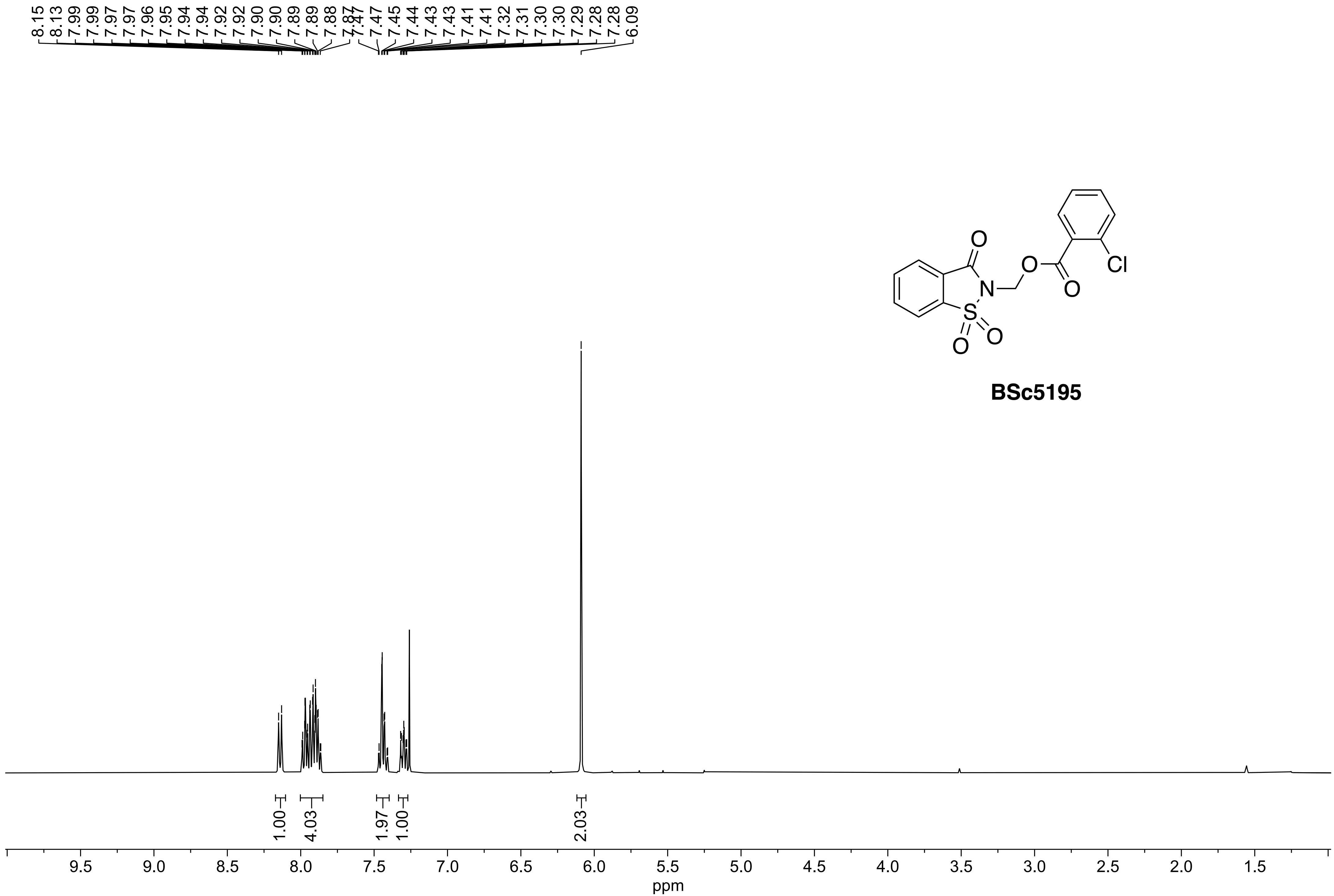

BSc5195

BSc5195

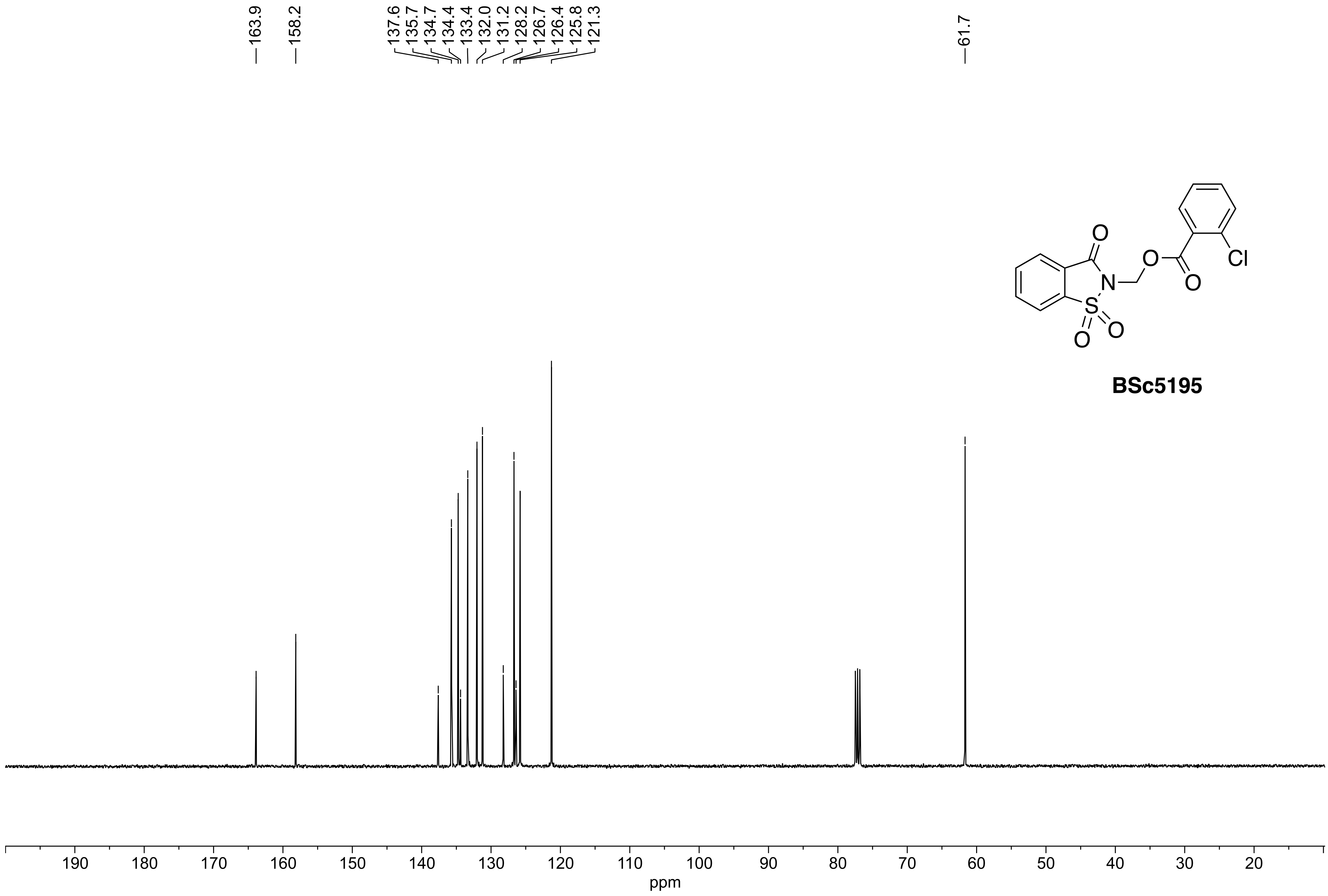

163.9
158.2
137.6
135.7
134.7
134.4
133.4
132.0
131.2
128.2
126.7
126.4
125.8
121.3
61.7
O
N
S
O
O
O
O
Cl
BSc5195
190
180
170
160
150
140
130
120
110
100
90
80
70
60
50
40
30
20
ppm

GNM01

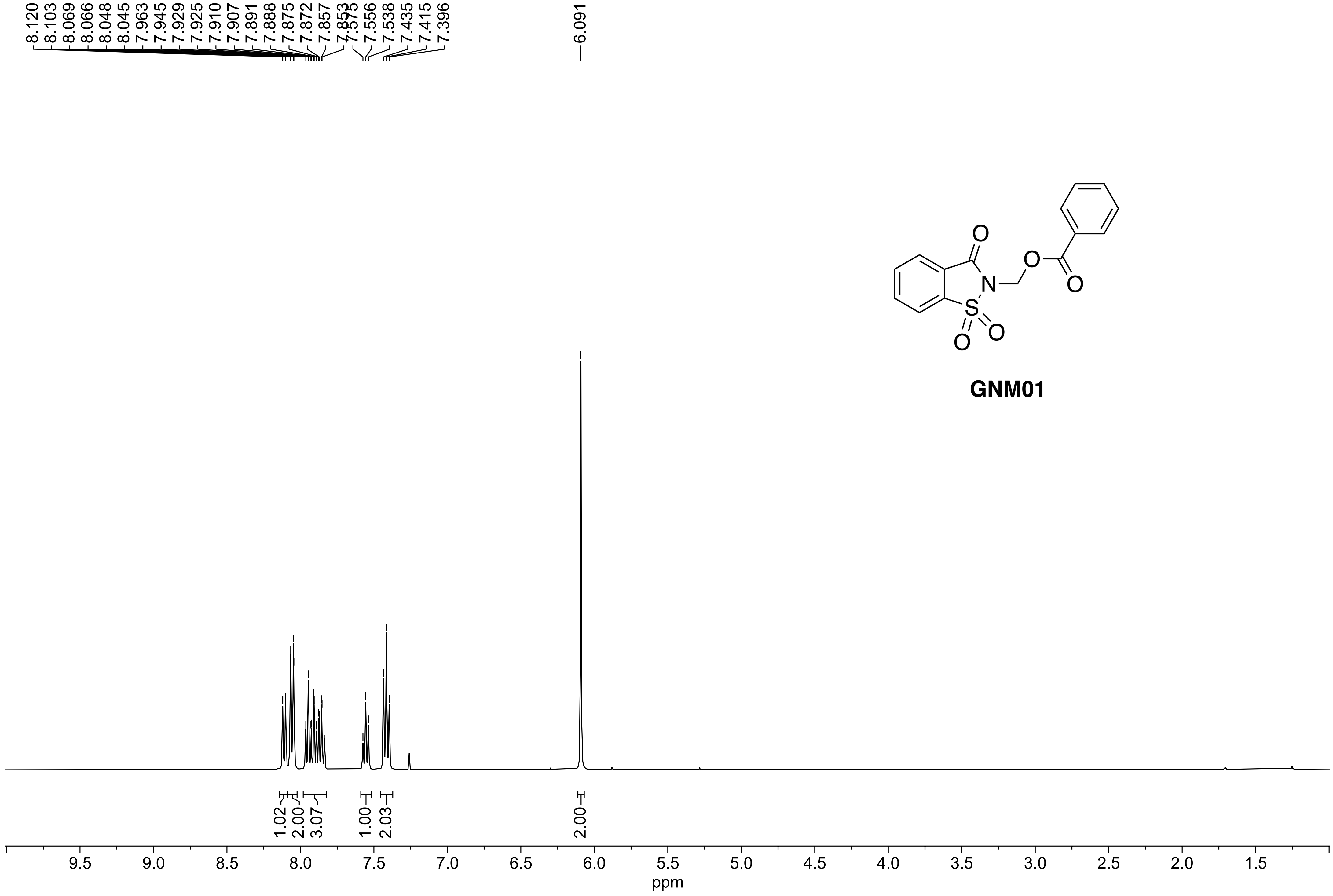
8.120
8.103
8.069
8.066
8.048
8.045
7.963
7.945
7.929
7.925
7.910
7.907
7.891
7.888
7.875
7.872
7.857
7.853
7.575
7.556
7.538
7.435
7.415
7.396
6.091
1.02
2.00
3.07
1.00
2.03
2.00
9.5
9.0
8.5
8.0
7.5
7.0
6.5
6.0
5.5
5.0
4.5
4.0
3.5
3.0
2.5
2.0
1.5
ppm
O
N
S
O
O
O
O

GNM01

GNM01

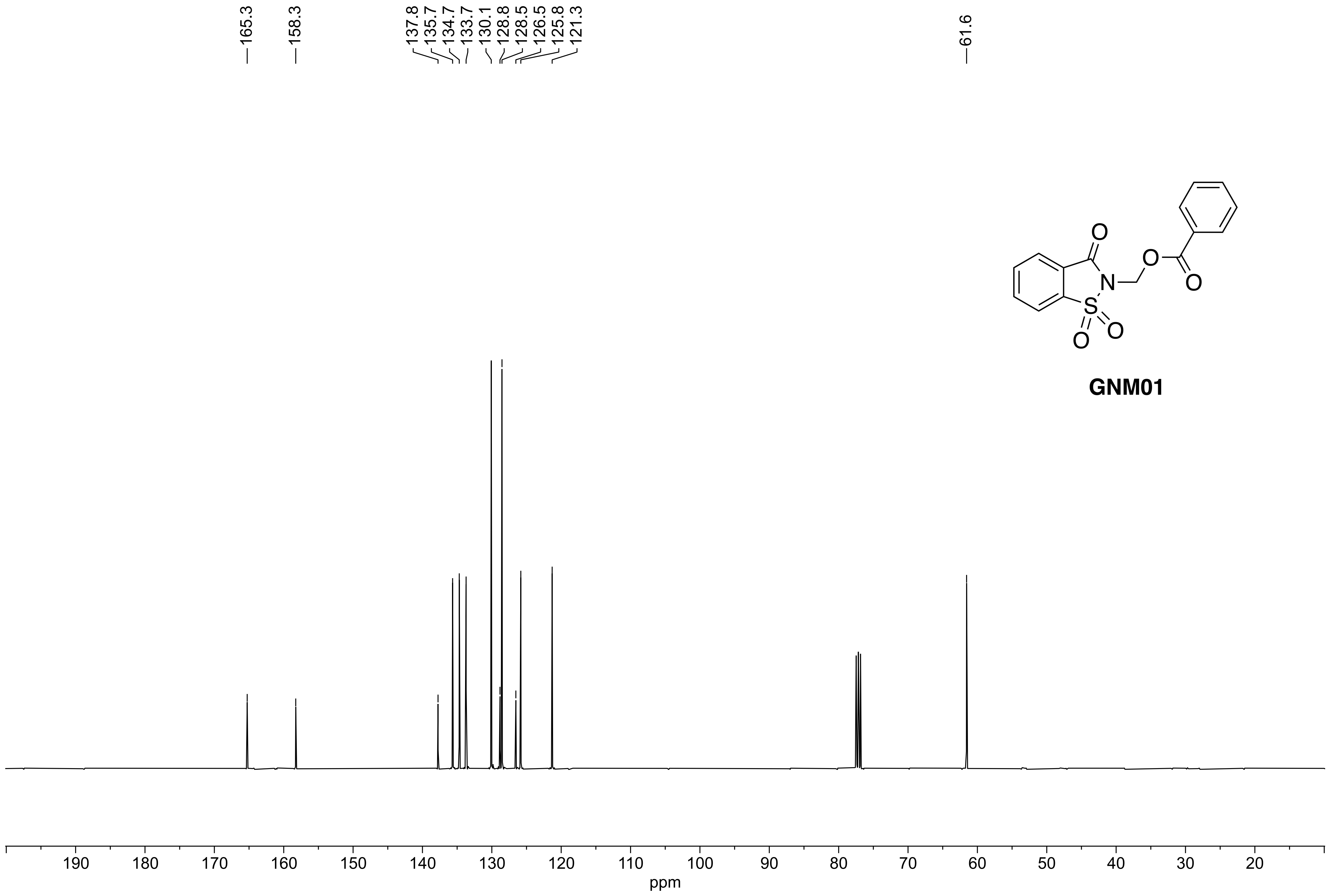
165.3
158.3
137.8
135.7
134.7
133.7
130.1
128.8
128.5
126.5
125.8
121.3
61.6
190
180
170
160
150
140
130
120
110
100
90
80
70
60
50
40
30
20
ppm
O
N
S
O
O
O
O

GNM01

GNM02

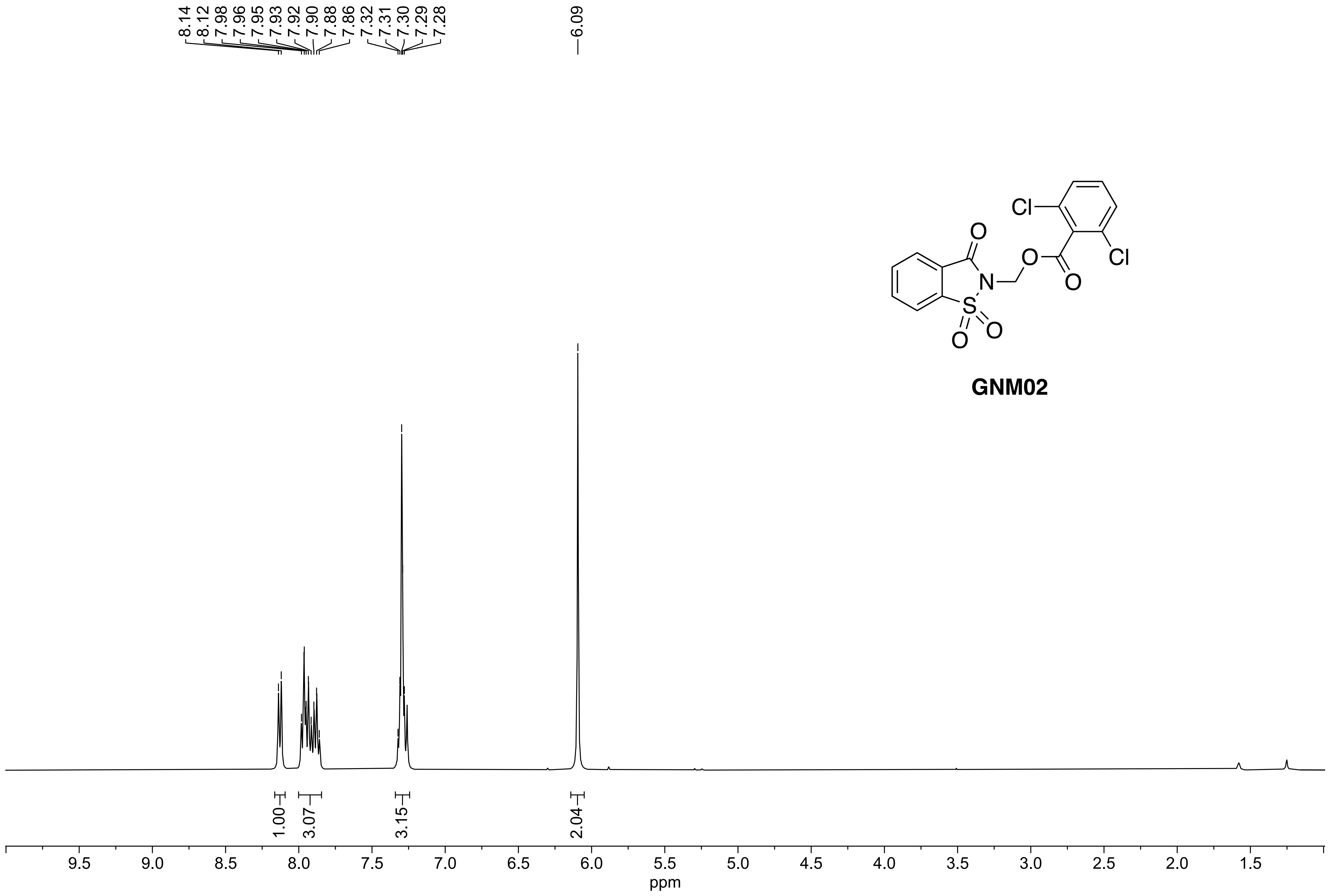

8.14
8.12
7.98
7.96
7.95
7.93
7.92
7.90
7.88
7.86
7.32
7.31
7.30
7.29
7.28
6.09
Cl
O
O
N
Cl
S
O
O
O
1.00
3.07
3.15
2.04
9.5
9.0
8.5
8.0
7.5
7.0
6.5
6.0
5.5
5.0
4.5
4.0
3.5
3.0
2.5
2.0
1.5
ppm

GNM02

GNM02

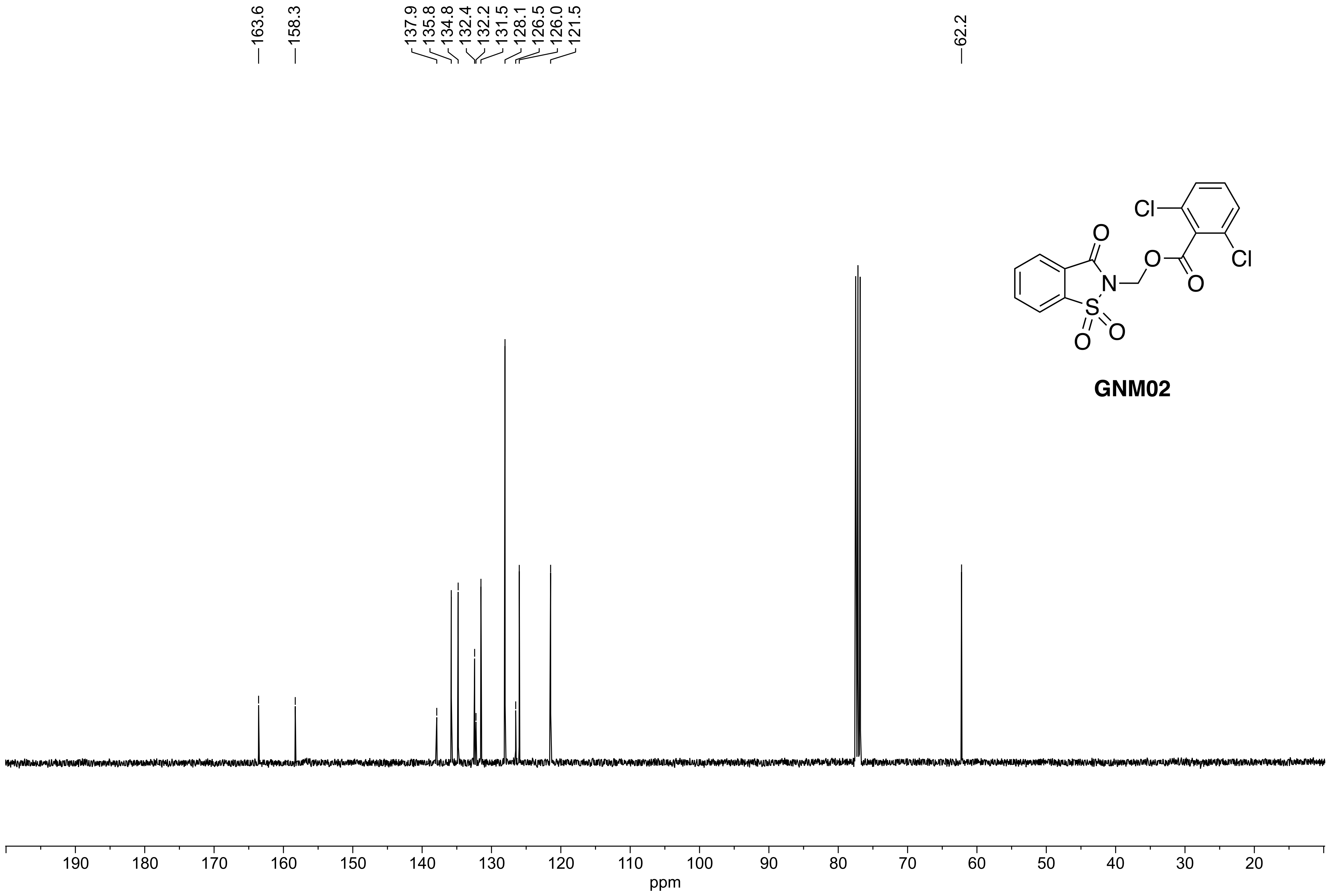
163.6
158.3
137.9
135.8
134.8
132.4
132.2
131.5
128.1
126.5
126.0
121.5
62.2
190
180
170
160
150
140
130
120
110
100
90
80
70
60
50
40
30
20
ppm
Cl
O
O
N
Cl
O
S
O
O

GNM02

GNM03

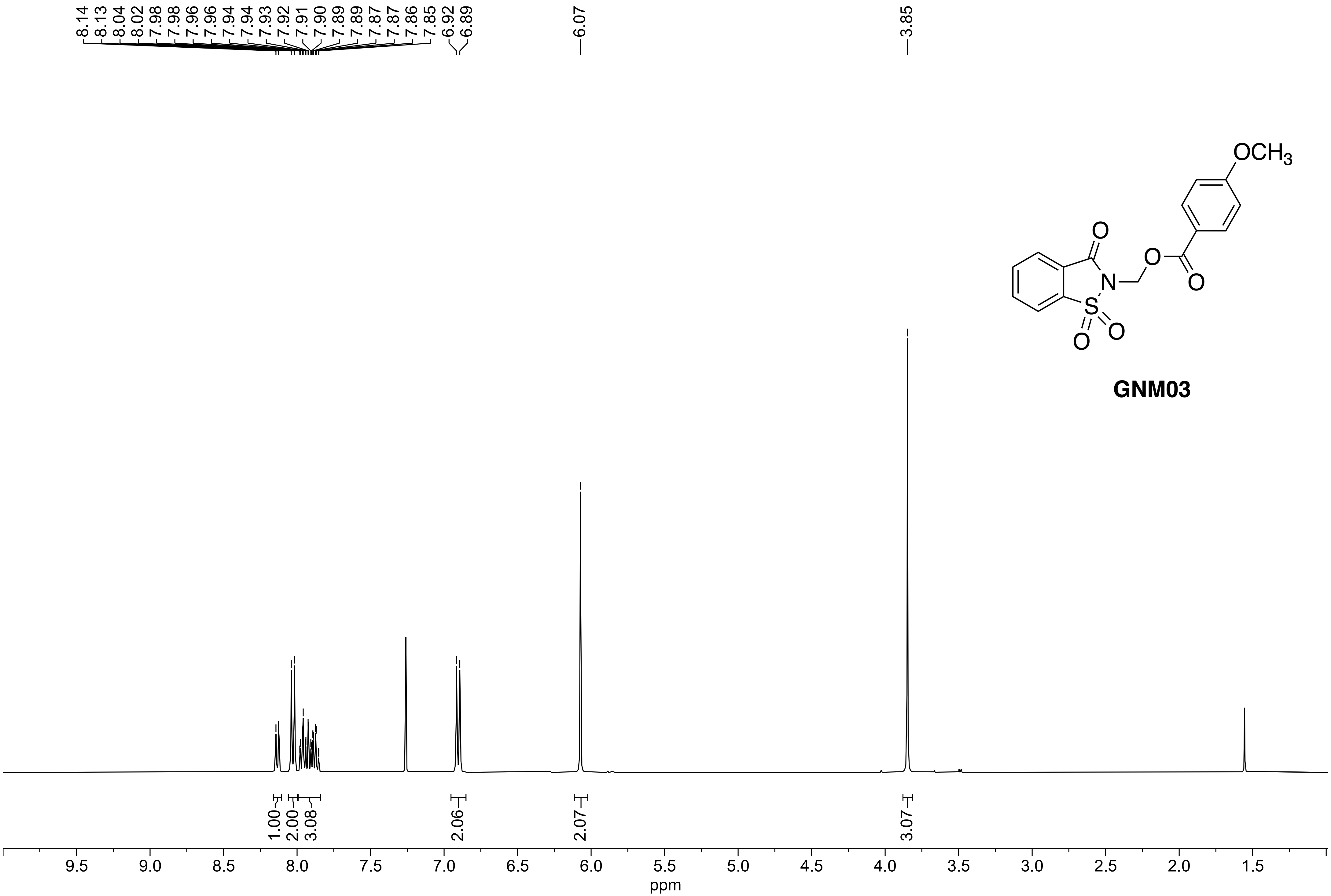
8.14
8.13
8.04
8.02
7.98
7.98
7.96
7.96
7.94
7.94
7.93
7.92
7.91
7.90
7.89
7.89
7.87
7.87
7.86
7.85
6.92
6.89
6.07
3.85
OCH3
O
N
O
O
S
O
O
1.00
2.00
3.08
2.06
2.07
3.07
9.5
9.0
8.5
8.0
7.5
7.0
6.5
6.0
5.5
5.0
4.5
4.0
3.5
3.0
2.5
2.0
1.5
ppm

GNM03

GNM03

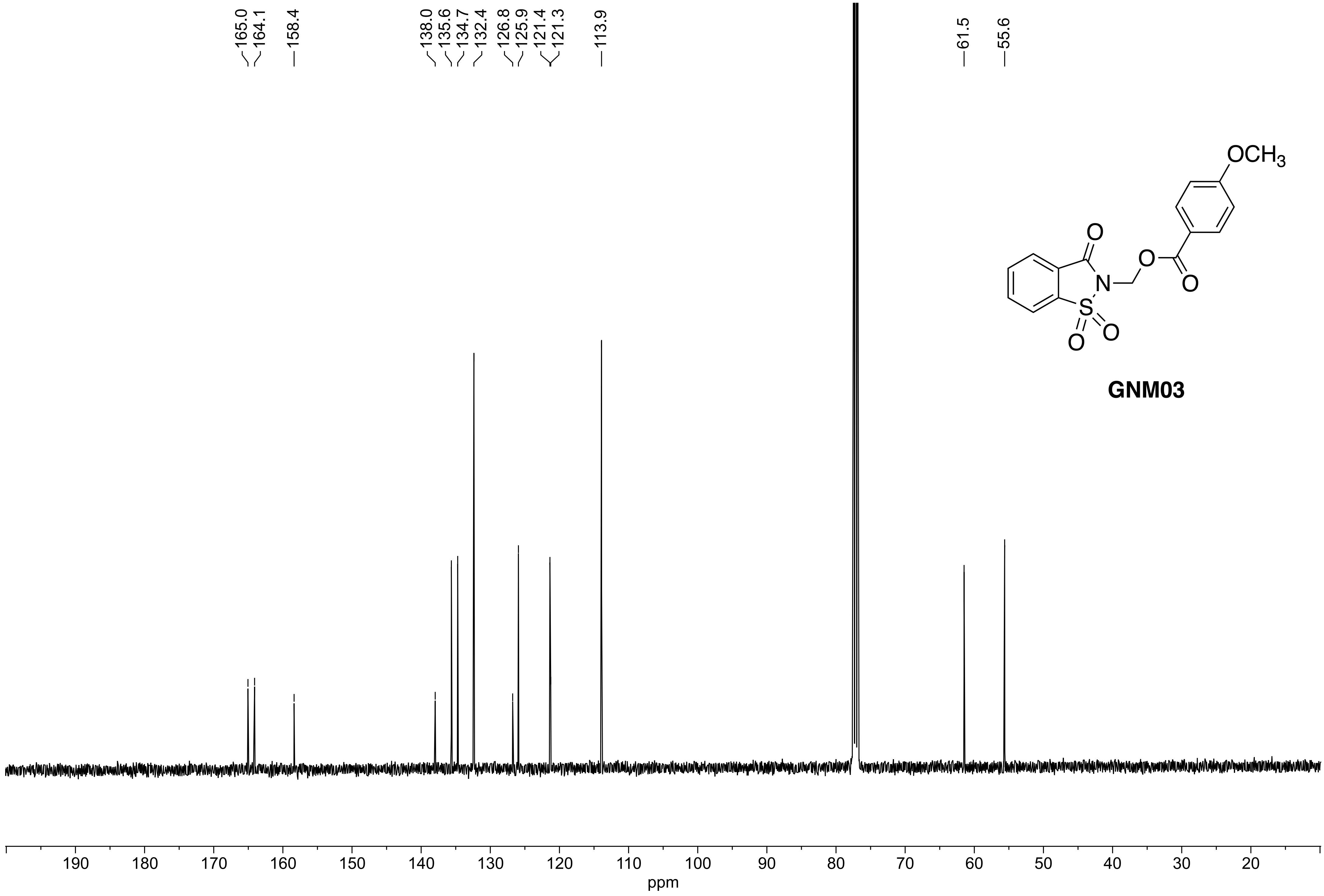

165.0
164.1
158.4
138.0
135.6
134.7
132.4
126.8
125.9
121.4
121.3
113.9
61.5
55.6
OCH_3
190
180
170
160
150
140
130
120
110
100
90
80
70
60
50
40
30
20
ppm

GNM03

GNM04

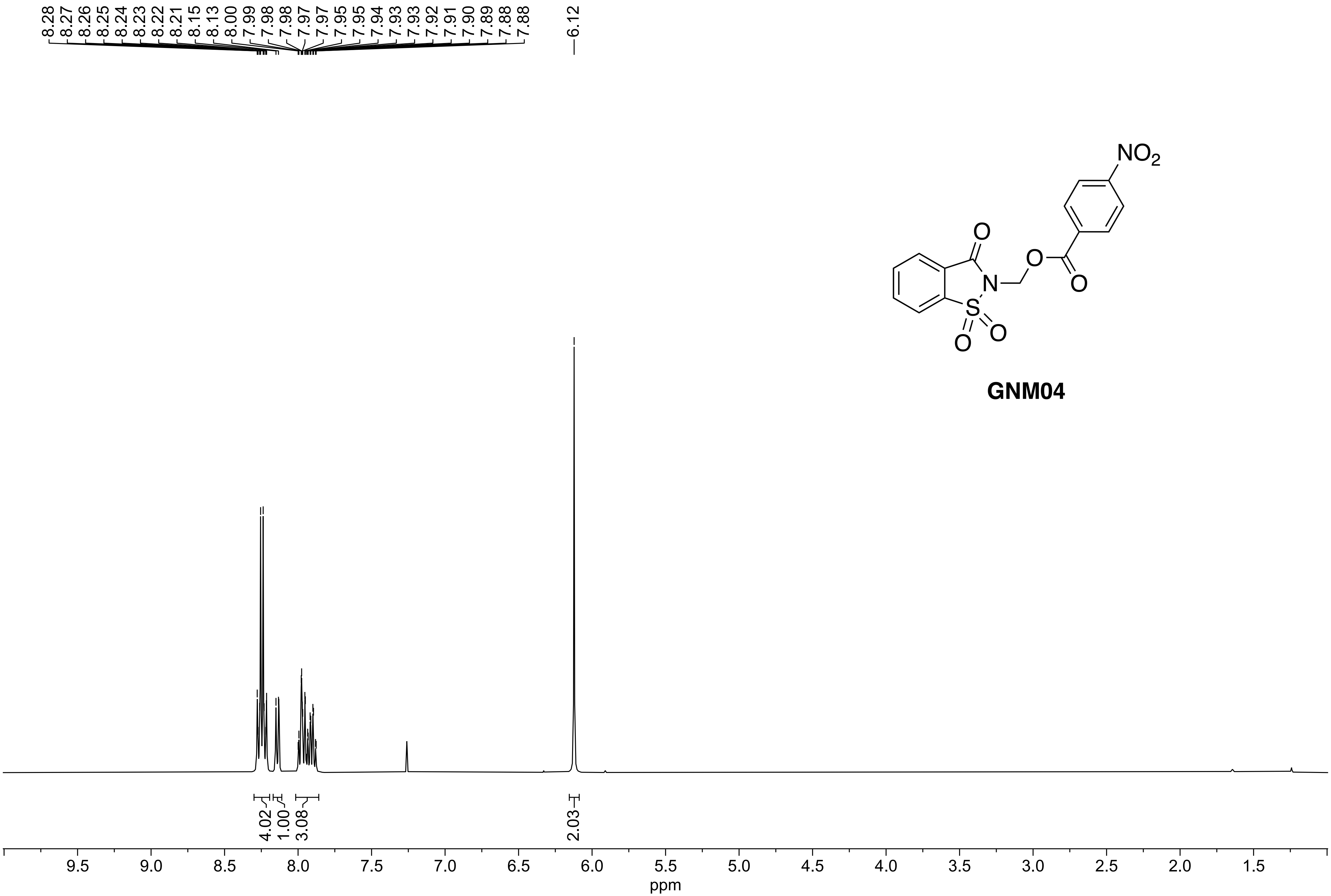
8.28
8.27
8.26
8.25
8.24
8.23
8.22
8.21
8.15
8.13
8.00
7.99
7.98
7.98
7.97
7.97
7.95
7.95
7.94
7.93
7.93
7.92
7.91
7.90
7.89
7.88
7.88
6.12
4.02
1.00
3.08
2.03
9.5
9.0
8.5
8.0
7.5
7.0
6.5
6.0
5.5
5.0
4.5
4.0
3.5
3.0
2.5
2.0
1.5
ppm
NO2
O
O
N
S
O
O
O

GNM04

GNM04

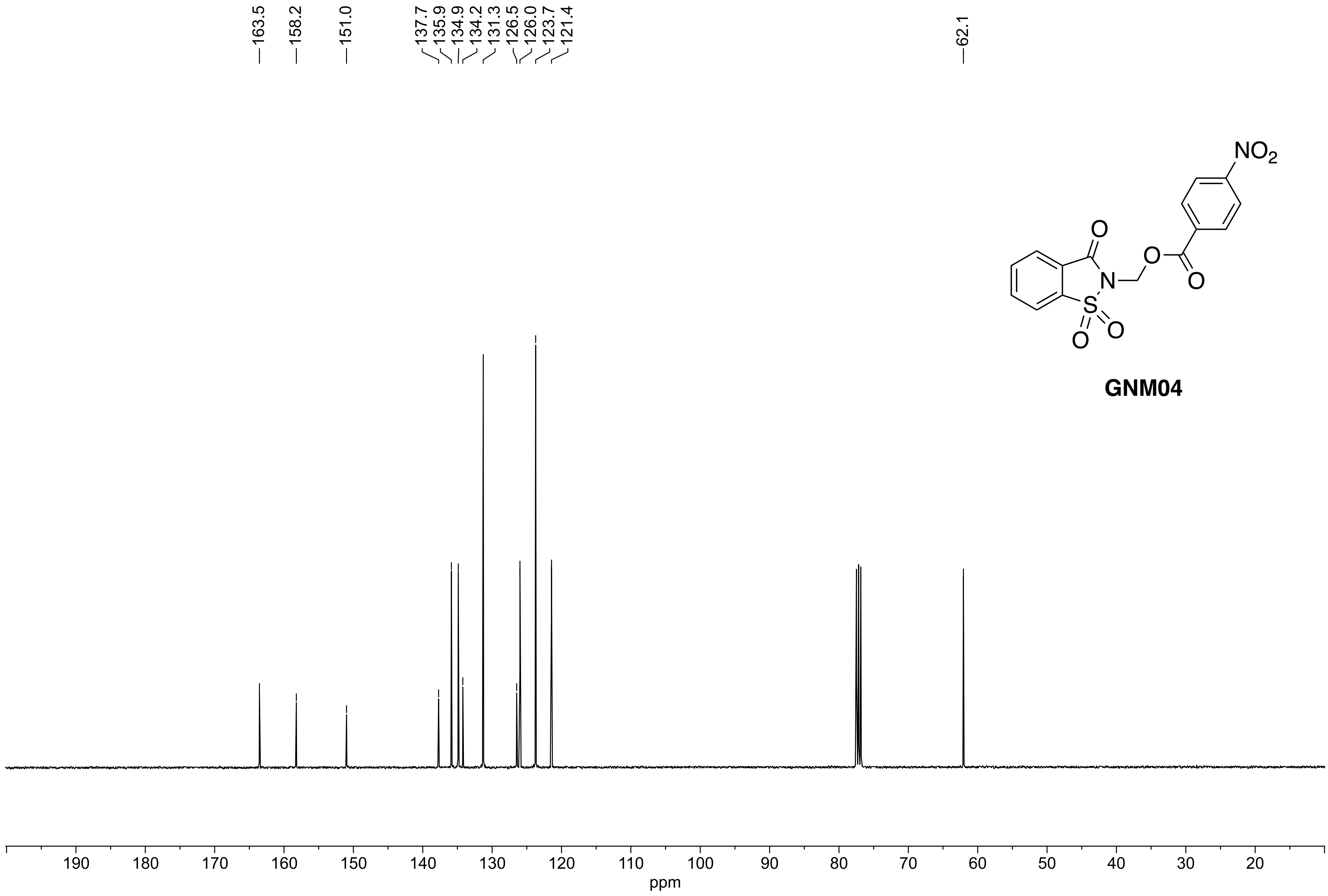
163.5
158.2
151.0
137.7
135.9
134.9
134.2
131.3
126.5
126.0
123.7
121.4
62.1
NO_2
GNM04
190
180
170
160
150
140
130
120
110
100
90
80
70
60
50
40
30
20
ppm

GNM05

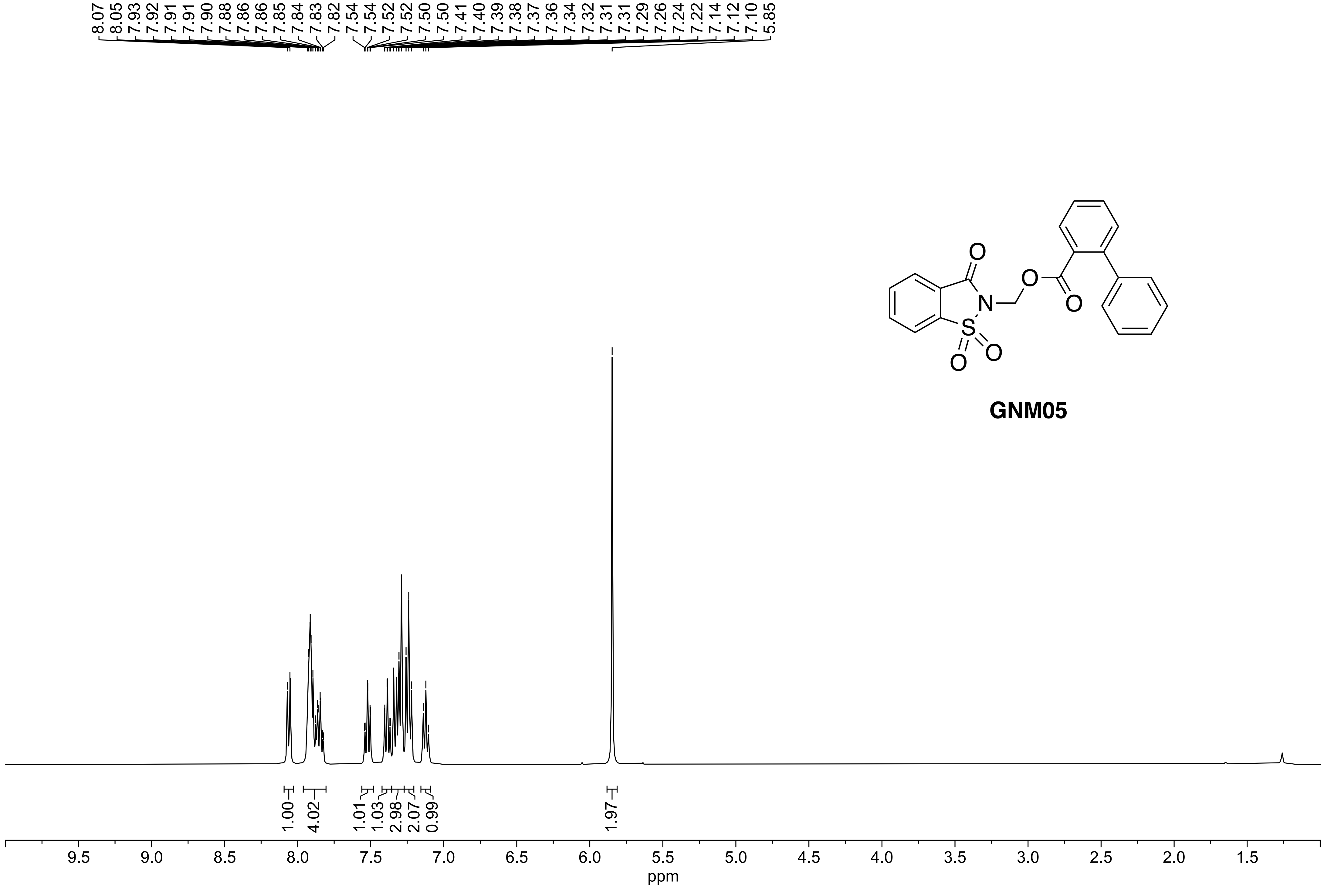
8.07
8.05
7.93
7.92
7.91
7.91
7.90
7.88
7.86
7.86
7.85
7.84
7.83
7.82
7.54
7.54
7.52
7.52
7.50
7.50
7.41
7.40
7.39
7.38
7.37
7.36
7.34
7.32
7.31
7.31
7.29
7.26
7.24
7.22
7.14
7.12
7.10
5.85
O
N
S
O
O
O
O
1.00
4.02
1.01
1.03
2.98
2.07
0.99
1.97
9.5
9.0
8.5
8.0
7.5
7.0
6.5
6.0
5.5
5.0
4.5
4.0
3.5
3.0
2.5
2.0
1.5
ppm

GNM05

GNM05

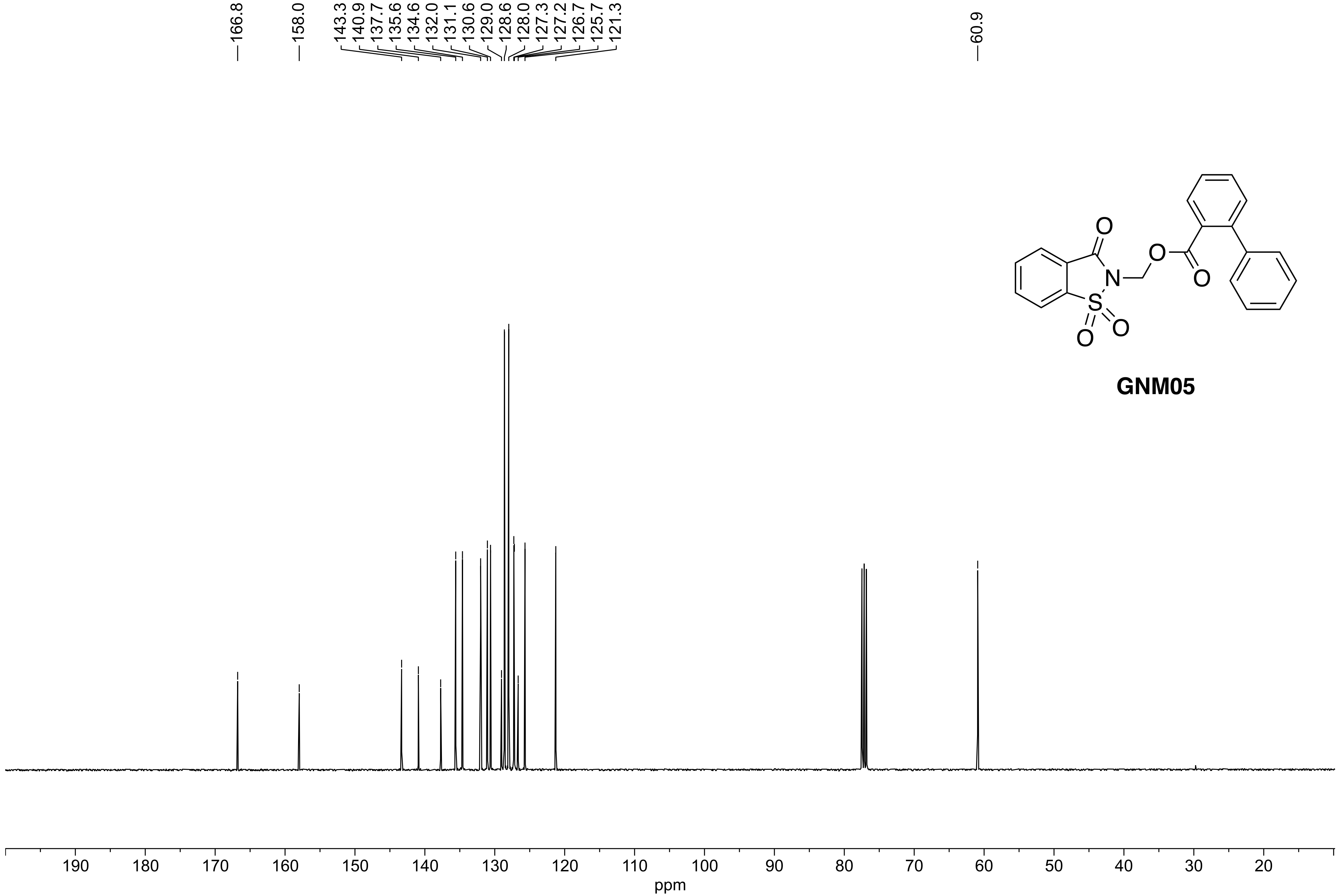

166.8
158.0
143.3
140.9
137.7
135.6
134.6
132.0
131.1
130.6
129.0
128.6
128.0
127.3
127.2
126.7
125.7
121.3
60.9
190
180
170
160
150
140
130
120
110
100
90
80
70
60
50
40
30
20
ppm
O
N
O
O
S
O
O

GNM05

GNM06

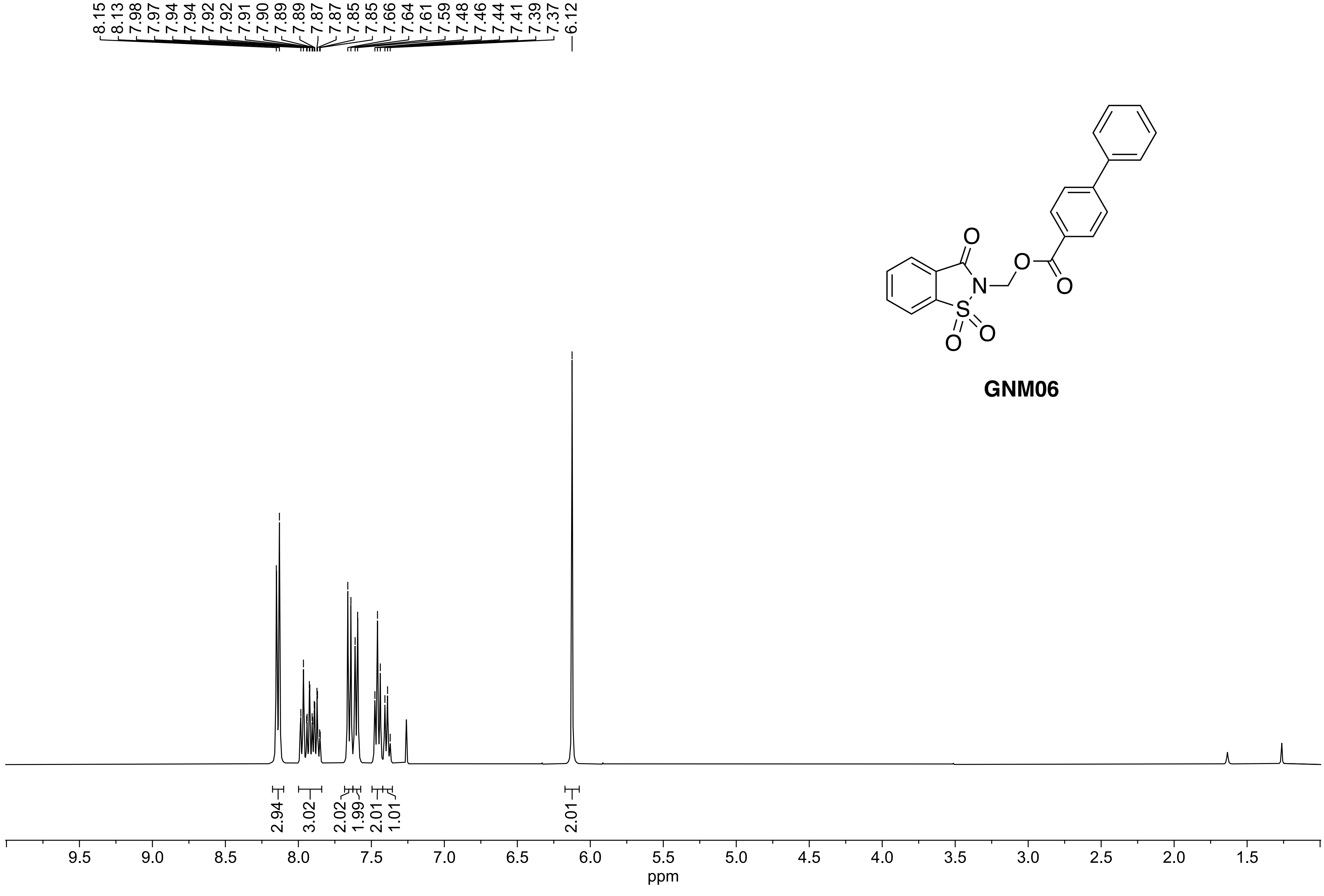
8.15
8.13
7.98
7.97
7.94
7.94
7.92
7.92
7.91
7.90
7.89
7.89
7.87
7.87
7.85
7.85
7.66
7.64
7.61
7.59
7.48
7.46
7.44
7.41
7.39
7.37
6.12
2.94
3.02
2.02
1.99
2.01
1.01
2.01
9.5
9.0
8.5
8.0
7.5
7.0
6.5
6.0
5.5
5.0
4.5
4.0
3.5
3.0
2.5
2.0
1.5
ppm
O
N
S
O
O
O
O

GNM06

GNM06

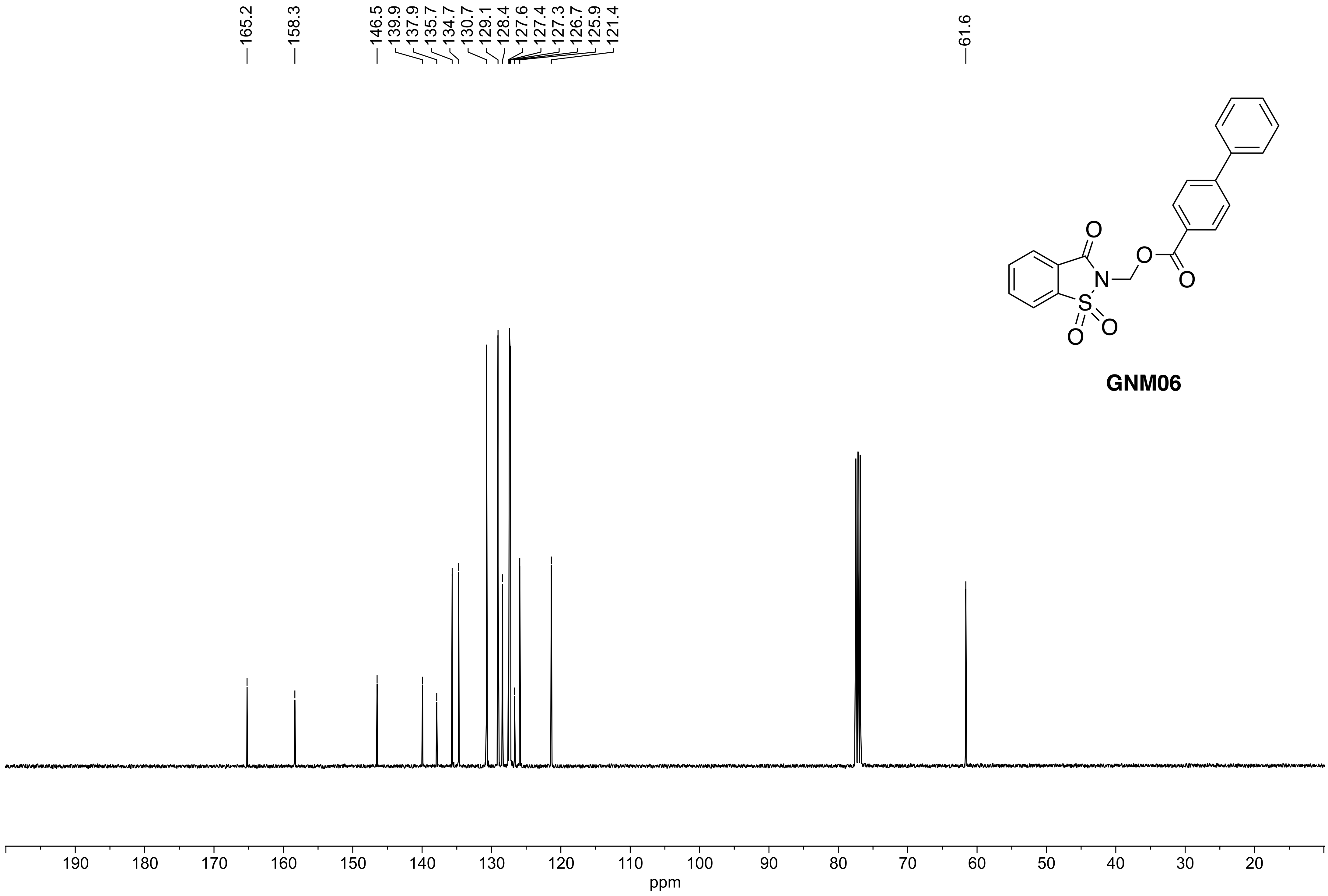

165.2
158.3
146.5
139.9
137.9
135.7
134.7
130.7
129.1
128.4
127.6
127.4
127.3
126.7
125.9
121.4
61.6
O
N
S
O
O
O
O
190
180
170
160
150
140
130
120
110
100
90
80
70
60
50
40
30
20
ppm

GNM06

GNM07

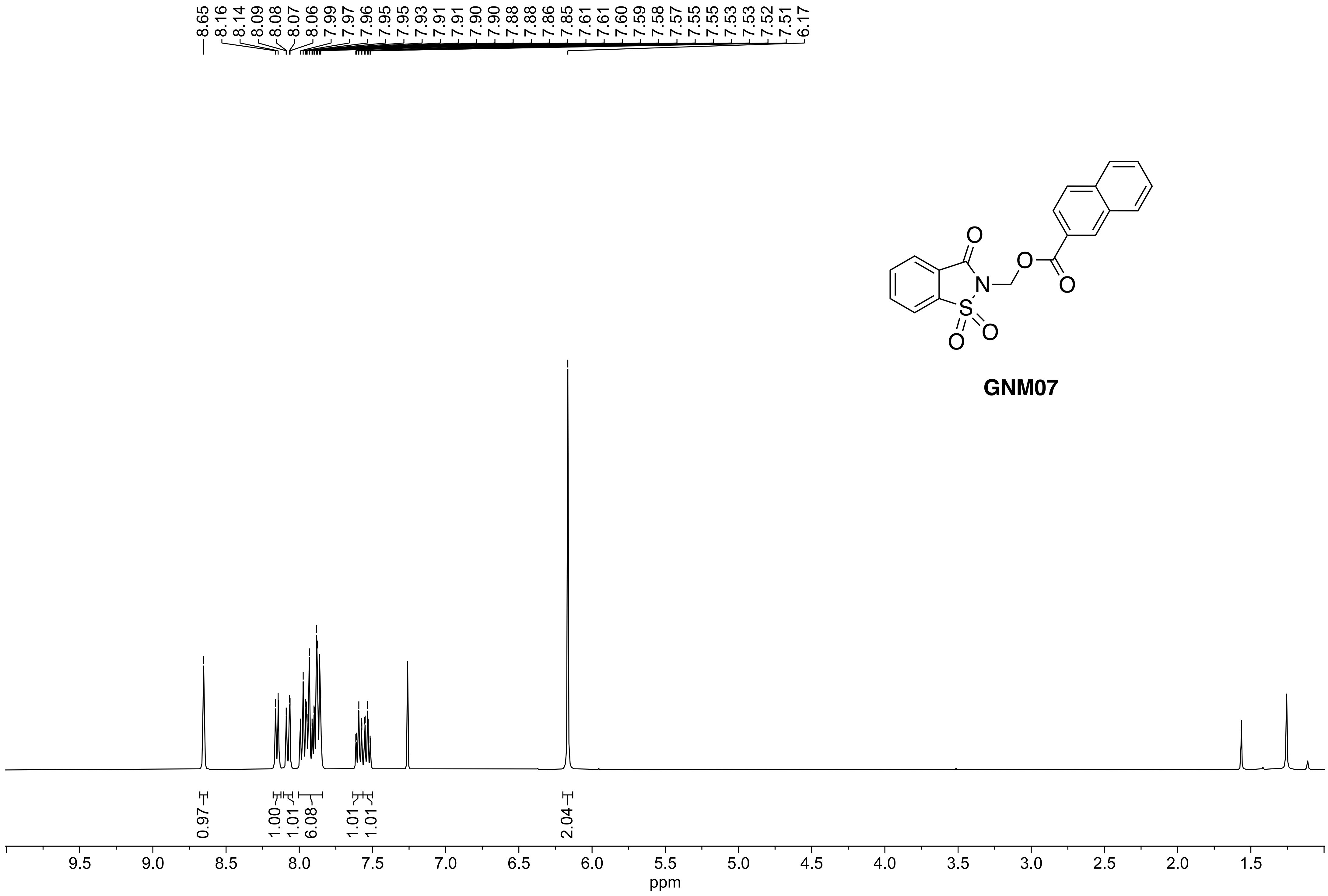
8.65
8.16
8.14
8.09
8.08
8.07
8.06
7.99
7.97
7.96
7.95
7.95
7.93
7.91
7.91
7.90
7.90
7.88
7.88
7.86
7.85
7.61
7.61
7.60
7.59
7.58
7.57
7.55
7.55
7.53
7.53
7.52
7.51
6.17
0.97
1.00
1.01
6.08
1.01
1.01
2.04
9.5
9.0
8.5
8.0
7.5
7.0
6.5
6.0
5.5
5.0
4.5
4.0
3.5
3.0
2.5
2.0
1.5
ppm
O
N
S
O
O
O
O

GNM07

GNM07

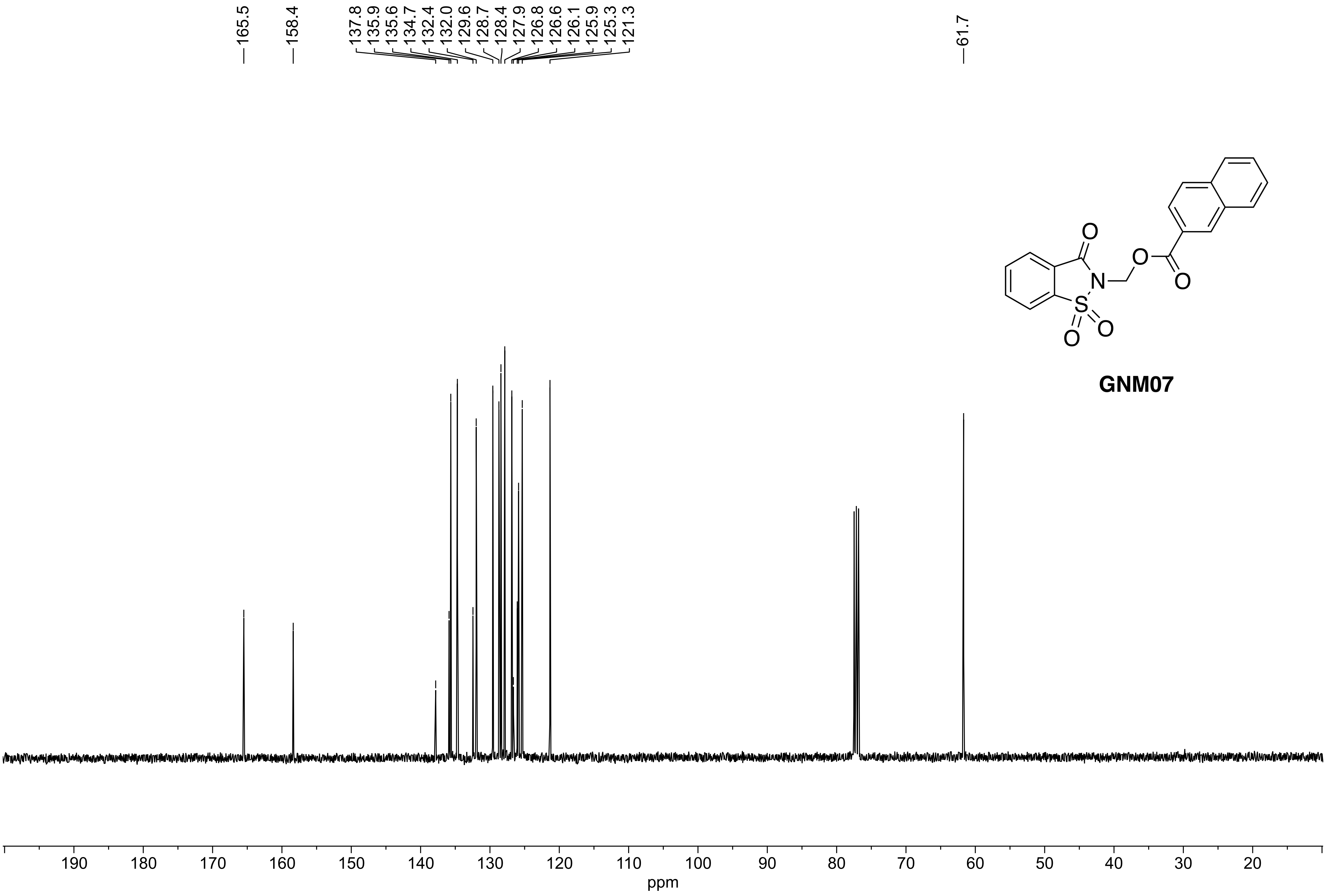

165.5
158.4
137.8
135.9
135.6
134.7
132.4
132.0
129.6
128.7
128.4
127.9
126.8
126.6
126.1
125.9
125.3
121.3
61.7
GNM07
190 180 170 160 150 140 130 120 110 100 90 80 70 60 50 40 30 20
ppm
